# Isoniazid or rifampicin preventive therapy with and without screening for subclinical TB: a modeling analysis

**DOI:** 10.1186/s12916-021-02189-w

**Published:** 2021-12-14

**Authors:** Emily A. Kendall, Hamidah Hussain, Amber Kunkel, Rachel W. Kubiak, Anete Trajman, Richard Menzies, Paul K. Drain

**Affiliations:** 1 Division of Infectious Diseases and Center for Tuberculosis Research, Johns Hopkins University School of Medicine, 1550 Orleans Street, Baltimore, Maryland 21287 USA; 2 Interactive Research and Development (IRD) Global, 583 Orchard Road #06-01 Forum, Singapore, Singapore; 3 Emerging Diseases Epidemiology Unit, Institut Pasteur, 25-28 Rue du Dr Roux, 75015 Paris, France; 4 Department of Epidemiology, University of Washington, 3980 15th Ave NE, Seattle, Washington 98195 USA; 5 Instituto de Medicina Social, Universidade do Estado do Rio de Janeiro, R. São Francisco Xavier, Rio de Janeiro, 20550-900 Brazil; 6 Respiratory Epidemiology and Clinical Research Unit, Montreal Chest Institute & McGill International TB Centre, 3650 St-Urbain Street, Montreal, Quebec H2X 2P Canada; 7 Departments of Global Health, Medicine, and Epidemiology, University of Washington, Box 359927, 325 Ninth Ave, Seattle, Washington 98104 USA

**Keywords:** Tuberculosis infection, Subclinical tuberculosis, Preventive therapy, Screening, Chest radiography, Global health, Antimicrobial resistance

## Abstract

**Background:**

Short-course, rifamycin-based regimens could facilitate scale-up of tuberculosis preventive therapy (TPT), but it is unclear how stringently tuberculosis (TB) disease should be ruled out before TPT use.

**Methods:**

We developed a state-transition model of a TPT intervention among two TPT-eligible cohorts: adults newly diagnosed with HIV in South Africa (PWH) and TB household contacts in Pakistan (HHCs). We modeled two TPT regimens—4 months of rifampicin [4R] or 6 months of isoniazid [6H]—comparing each to a reference of no intervention. Before initiating TPT, TB disease was excluded either through symptom-only screening or with additional radiographic screening that could detect subclinical TB but might limit access to the TPT intervention. TPT’s potential curative effects on both latent and subclinical TB were modeled, as were both acquisitions of resistance and prevention of drug-resistant disease. Although all eligible individuals received the screening and/or TPT interventions, the modeled TB outcomes comprised only those with latent or subclinical TB that would have progressed to symptomatic disease if untreated.

**Results:**

When prescribed after only symptom-based TB screening (such that individuals with subclinical TB were included among TPT recipients), 4R averted 45 active (i.e., symptomatic) TB cases (95% uncertainty range 24–79 cases or 40–89% of progressions to active TB) per 1000 PWH [17 (9–29, 43–94%) per 1000 HHCs]; 6H averted 37 (19–66, 52–73%) active TB cases among PWH [13 (7–23, 53–75%) among HHCs]. With this symptom-only screening, for each net rifampicin resistance case added by 4R, 12 (3–102) active TB cases were averted among PWH (37 [9–580] among HHCs); isoniazid-resistant TB was also reduced. Similarly, 6H after symptom-only screening increased isoniazid resistance while reducing overall and rifampicin-resistant active TB. Screening for subclinical TB before TPT eliminated this net increase in resistance to the TPT drug; however, if the screening requirement reduced TPT access by more than 10% (the estimated threshold for 4R among HHCs) to 30% (for 6H among PWH), it was likely to reduce the intervention’s overall TB prevention impact.

**Conclusions:**

All modeled TPT strategies prevent TB relative to no intervention, and differences between TPT regimens or between screening approaches are small relative to uncertainty in the outcomes of any given strategy. If most TPT-eligible individuals can be screened for subclinical TB, then pairing such screening with rifamycin-based TPT maximizes active TB prevention and does not increase rifampicin resistance. Where subclinical TB cannot be routinely excluded without substantially reducing TPT access, the choice of TPT regimen requires weighing 4R’s efficacy advantages (as well as its greater safety and shorter duration that we did not directly model) against the consequences of rifampicin resistance in a small fraction of recipients.

**Supplementary Information:**

The online version contains supplementary material available at 10.1186/s12916-021-02189-w.

## Background

Tuberculosis (TB) preventive therapy (TPT) reduces TB incidence and mortality [1, 2] and is considered an essential tool for ending the TB pandemic [3–5]. Short-course TPT regimens could improve the global implementation and effectiveness of TPT [6]. Isoniazid for at least 6 months (6H) has long been standard, but shorter, rifamycin-based regimens—including 4 months of daily rifampicin (4R) or 3 months of weekly rifapentine and isoniazid (3HP)—are efficacious, better tolerated, and more cost-effective [7–10] and have been included in recent TB prevention guidelines [11, 12].

TPT has not been observed to cause clinically significant increases in drug resistance [13, 14], but the available data come from studies that rigorously excluded TB disease before prescribing TPT. Thus, concern about the potential consequences of inadvertent monotherapy remains a barrier to access to TPT in general [15], and to rifamycin-based TPT in particular given the importance of rifamycins in the treatment of active TB [16, 17]. Requirements that TB disease be excluded with sensitive radiographic or bacteriologic testing before TPT may, however, limit access [18–20]. Recognizing this, WHO guidance only conditionally recommends chest radiographic screening for TPT-eligible adult contacts and offers it as an option for people living with HIV (PWH) who are receiving antiretroviral therapy; guideline advise that decisions be guided by “local epidemiology, health infrastructure, and resources” [11].

The relevant tradeoffs between sensitively excluding TB disease and maximizing TPT access have not been quantified in a way that can guide clinicians and program managers, nor have they compared between isoniazid- and rifamycin-based TPT regimens. To address this need, we developed a decision analytic model of TB screening and TPT in two populations for whom TPT is recommended [11]: PWH and household contacts (HHCs). We compared outcomes after symptom-only TB screening and after radiographic screening (with, potentially, a reduction in TPT access when radiography was required) for two TPT regimens: 6 months of isoniazid (6H, still the most widely used regimen globally [21]) and 4R (the regimen with the greatest potential for acquired rifampicin resistance, and also a conservative proxy for 3HP).

## Methods

### TPT simulation model

We constructed state-transition models of a PWH cohort of 1000 adults newly diagnosed with HIV and initiating antiretroviral therapy (ART) and a HHC cohort of 1000 all-age household contacts [22, 23] of recently diagnosed drug-susceptible TB index patients. Each cohort was stratified by TB state, drug susceptibility (pan-susceptible, isoniazid monoresistant, rifampicin monoresistant, or multidrug [isoniazid and rifampicin] resistant), and either CD4 count (for PWH) or age (for HHCs). TB states included active disease (symptomatic), subclinical progressors (currently asymptomatic but microbiologically or radiographically detectable, and on a course that would progress to active if untreated), latent progressors (with infections that would progress to active disease at some time in the future), and those who would never progress to active disease. We modeled an intervention of TPT (4R or 6H) with or without a test to first exclude subclinical TB).

**Fig. 1:**
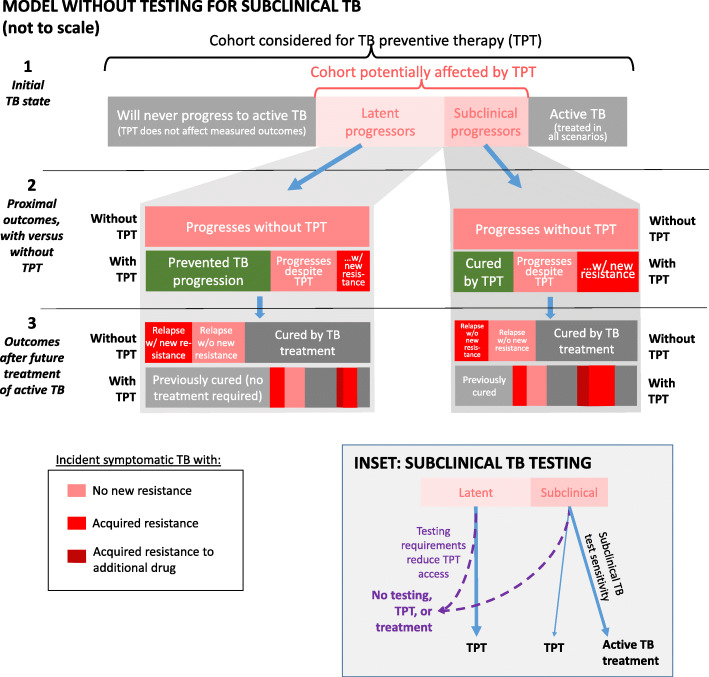
Conceptual overview of the model, without screening for subclinical TB (main panel) and with screening (inset). Within each cohort, we identify two subgroups whose outcomes may be affected by TPT: those with a latent infection that will, if untreated, progress to active TB at some point in the future (“latent progressors”), and those who have prevalent subclinical TB (asymptomatic but detectable through additional testing such as chest X-ray or sputum testing) that will progress to active rather than resolving spontaneously (“subclinical progressors”). These subgroups are further stratified (not shown) by the drug resistance present at *t* = 0 and by CD4 count (people living with HIV) or age (household contacts). These latent and subclinical progressor groups are followed through the following steps: (1) testing to detect and treat subclinical TB in some modeled scenarios [inset], (2) TPT (if given), and (3) TB treatment (if subclinical TB is diagnosed, or if progression to active TB occurs despite the intervention). For the TPT and treatment steps, the probabilities of cure, and of acquiring new or additional resistance if not cured, depend on the regimen and the drug resistance present. When testing for subclinical TB is required prior to TPT (Inset), it reduces access (such that a proportion of individuals with latent and subclinical TB receive neither the additional testing nor TPT), but a proportion of the remaining individuals with subclinical TB are identified and immediately treated for active TB. Proportions in the figure are not to scale. All modeled scenarios include symptom screening to detect and treat active TB, so the outcomes of those with active (symptomatic) TB at enrollment are not compared between scenarios

To focus on outcomes that TPT could affect, we simulated two distinct subgroups in progressive TB states within each cohort, illustrated in Fig. 1:

1. “Latent progressors,” with *M. tuberculosis* infection but no current active TB disease, whose infections would, in absence of TPT, eventually progress to active disease;
2. “Subclinical progressors” with TB disease that was undetectable by a symptom screen but was microbiologically active (e.g., culture-positive) and would progress to symptoms (i.e., to active disease) if untreated.

Additional individuals with latent infections that would never progress to active disease, or with a prevalent subclinical disease that would resolve even without intervention, were not included in these “progressor” subgroups.

For these subgroups, we simulated the following steps (Fig. 1):

1. Symptom screening: At the time of contact investigation or antiretroviral therapy initiation, all individuals were screened for active (i.e., symptomatic) TB disease. (Those who had a positive symptom screen and were confirmed to have ﻿active TB disease were assumed to receive treatment under all scenarios and were not modeled further.)
2. Possible additional screening (Fig. 1 inset): For individuals without symptoms, either (a) all were offered TPT or (b) they underwent an additional screening test such as chest radiography (assumed 90% sensitive for all subclinical TB, regardless of whether it would progress if untreated). Such screening, if required, could be unavailable to a proportion of the cohort (20% in primary analysis), who was then not eligible for TPT.
3. TPT for individuals not diagnosed with TB disease (Fig. 1, section 2): If latent progressors or subclinical progressors received TPT, they could be cured (more likely for latent than for subclinical TB) or they could progress to active disease with or without acquisition of resistance. TPT was assumed to have no effect if pre-existing resistance to the TPT drug was present.
4. Treatment of TB disease when indicated (Fig. 1, section 3): For subclinical progressors who were diagnosed and treated at the time of TPT consideration, and also for individuals who progressed from latent or subclinical to active TB in the future and were treated at that later time, we modeled the outcomes of TB treatment (Additional file 1: Table S4 [24–32]). Treatment failures and relapses were counted as active TB episodes when estimating the impact of TPT interventions on future TB incidence and drug resistance. Thus, we captured TPT’s downstream effects on active TB incidence and drug resistance outcomes through the prevention of TB episodes whose treatment may have otherwise resulted in failure, relapse, or acquired resistance. Individuals with subclinical TB that would have resolved without intervention were not modeled as being at risk for treatment failure or relapse, even if they were treated.

### Parameter estimation

#### Cohort composition

Key model parameters are shown in Table 1, with a full description in Additional file 1: Supplemental Methods [7, 8, 13, 21, 24–74].

**Table 1:** Summary of model inputs (see Additional file 1 for details and numerical estimates)

Parameter type	Definition	Data sources and approach to estimation	Table in Additional file 1 with estimates, uncertainty, and references
Prevalence of “latent progressor” state among each cohort considered for TPT	Latent infections that will progress to active (symptomatic) TB disease at some time in the future	Lifetime cumulative incidence extrapolated from observed 12-month incidence (PWH cohort) or baseline prevalence (household contacts) using published cohort studies and meta-analyses	Table S1
Prevalence of “subclinical progressor” state among each cohort considered for TPT	TB disease that is undetectable by a symptom screen but is microbiologically active and will eventually progress to active disease if untreated	Primary clinical data (Table 2): Symptom-negative individuals who progressed to active TB within 3 months (PWH) or who were diagnosed with TB during extensive baseline evaluation (household contacts, with adjustment for expected spontaneous resolution).	Table S1
Efficacy of TPT for latent progressors, by regimen	Proportion of latent progressions prevented, if initially susceptible to the TPT regimen and completes enough TPT to be at risk for acquired resistance	Network meta-analysis of clinical trial data, adjusted for reinfection, nonadherence, and baseline drug resistance. 6H efficacy parametrized relative to 4R and assumed equal or less than 4R.	Table S2
Reduction in TPT efficacy when used during subclinical TB	Proportion of TPT-preventable latent progressions that cannot be cured by TPT at the subclinical progressor stage	Bounded by the efficacy of TPT for latent TB and by the efficacy of monotherapy for symptomatic active TB.	Table S2
Risk of acquiring resistance to the TPT drug, if latent TB progresses despite TPT	Applies to those whose TPT is unsuccessful and whose initial infections were not drug-resistant.	Incidence of drug-resistant TB after TPT in clinical trials, adjusted for expected incidence from pre-existing drug resistance. Risk for isoniazid sets an upper bound on risk for rifampicin.	Table S3
Risk of acquiring resistance to the TPT drug, if subclinical TB progresses despite TPT	As above	Treatment trials with a single effective drug. Large uncertainty is reflected in wide parameter distributions.	Table S3
Outcomes after active TB treatment	Risk of failure/relapse, with or without acquired isoniazid or rifampicin resistance, as a function of initial susceptibilities.	Previous reviews of clinical trial and research cohort outcomes. Weighted based on the regimens expected to be used in present-day programmatic settings, including the use of first-line regimens when drug resistance goes undetected.	Table S4
Prevalence and overlap of INH and RIF resistance	Same for subclinical cases and latent progressors	Drug resistance survey data; lower in contacts of DS-TB patients than among all TB infections	Table S4
Baseline drug resistance	Prevalence and overlap of isoniazid and rifampicin resistance among TB infections in a modeled cohort.	National or regional drug resistance survey data, adjusted downward for household contacts of known DS-TB patients	Table S5

Abbreviations: *TPT* tuberculosis preventive treatment, *TB* tuberculosis, *PWH* patients newly diagnosed with HIV, *INH* isoniazid, *RIF* rifampicin, *DS* drug-susceptible

Primary clinical data, together with published systematic reviews, were used to estimate the number of latent progressors and subclinical progressors (i.e., people with TB infections or asymptomatic disease, respectively, that would later progress to active disease) within each cohort (Additional file 1: Supplemental Methods 1).

The PWH cohort is based on primary data from adults presenting for outpatient HIV testing in Kwa-Zulu Natal, South Africa, from September 2013 through February 2019 [75]. Enrollees were screened for TB based on symptoms, then followed for 12 months for incident TB. Patients who were initially asymptomatic but received diagnoses of TB within 3 months were assumed to have had progressive subclinical TB at enrollment; sputum culture of a representative subset of patients at enrollment [76] (Additional file 1: Table S6) supported this baseline prevalence estimate in a population among whom spontaneous resolution of subclinical TB is uncommon [77]. The prevalence of progressive subclinical disease was estimated within strata of baseline CD4 count (< 100, 100–200, 201–350, and > 350 cells/mm^3^), and the lifetime cumulative incidence of progression from latent infection to active TB disease, for infections present at enrollment, was extrapolated from the TB incidence observed in months 4 to 12 (Additional file 1: Supplemental Methods 2a).

The HHC cohort is based on a large TPT scale-up effort in Pakistan. All household contacts of drug-susceptible TB patients underwent a symptom screen, chest radiography, detailed clinical evaluation, and (if able to expectorate) sputum Xpert® MTB/RIF. Those with a negative symptom screen but a TB diagnosis were classified as having subclinical TB, and we estimated the proportion of these subclinical cases who would progress to active disease if untreated. We used pooled data from published household contact cohorts [39, 40] to estimate the future incidence of progression from latent to active disease, relative to the prevalence of TB disease at the time of contact investigation, stratified by age < 5, 5–15, or > 15 years (Additional file 1: Supplemental methods 2b).

The estimated prevalence of the subclinical progressor state was 3.5% (2.9–4.2%) among the newly diagnosed PWH and 0.4% (0.2–0.7%, based on 0.8% [0.7–1.0%] subclinical TB prevalence and 50% [30–70%] probability of progression) among the HHCs (Table 2). We estimated that there were between 0.2 and 1.2 subclinical progressors per latent progressor, depending on the cohort and the age or CD4 stratum (Table 2).

**Table 2:** Prevalence of active and subclinical TB, and estimated incidence of future progression from latent or subclinical to active TB, in primary data from patient cohorts in South Africa and Pakistan used to generate parameter inputs to the TPT model

	People newly diagnosed with HIV, Kwa-Zulu Natal, South Africa				Household contacts, Pakistan		
	CD4 < 100	CD4 100–200	CD4 200–350	CD4 > 350	Age < 5 years	Age 5–14 years	Age ≥ 15 years
**Total evaluated**	379	442	785	1392	2194	4261	6648
**Symptomatic TB at baseline, N (%)**	93 (24.5%)	56 (12.7%)	42 (5.4%)	43 (3.1%)	42 (1.9%)	86 (2%)	28 (0.4%)
**Subclinical TB diagnosed at baseline, N (%)**					19 (0.9%)	58 (1.4%)	29 (0.4%)
**Subclinical TB progressed to active within 3 months**	16 (4.2%)	17 (3.8%)	28 (3.6%)	44 (3.2%)			
**Followed to 6 months**	359	406	740	1312	NA	NA	NA
**TB diagnoses, 3 to 6 months, N (%**	2 (0.6%)	4 (1%)	2 (0.3%)	0 (0%)			
**Followed to 12 months**	314	348	625	1096	NA	NA	NA
**TB diagnoses, 6 to 12 months, N (%**	7 (2.2%)	7 (2%)	5 (0.8%)	7 (0.6%)			
**Estimated lifetime incidence (95%CI) of progression of latent infections present at enrollment**^**a**^	4.7% (2.7–6.8%)	4.9% (2.9–7.1%)	3.0% (1.7–4.2%)	2.5% (2.5–3.4%)	0.6% (0.3–1.0%)	3.7% (2.3–5.3%)	1.2% (0.8–1.8%)
**Estimated subclinical prevalent cases per future latent progression**					1.4	0.4	0.4
**Estimated subclinical progressors per latent progressor (95%CI)**^**b**^	0.9 (0.6–1.4)	0.8 (1.5–1.2)	1.2 (0.8–1.9)	1.2 (0.9–1.9)	0.7 (0.4–1.2)	0.2 (0.1–0.3)	0.2 (0.1–0.3)

^a^Estimates are derived by combining baseline and 12-month cohort outcomes with external data on the timing of TB progression and the balance of prevalence and incident TB in untreated household cohorts, as described in the Methods and Additional file 1

^b^For the household contact cohort, estimates account for the possibility that some subclinical TB will resolve without treatment rather than progressing to active TB. Uncertainty in the probability of spontaneous resolution is incorporated into the uncertainty in this parameter (Additional file 1: Table S2)

#### TPT outcome parameters

In clinical trials of TPT [7, 8, 13, 59], and in programmatic settings with well-documented outcomes [78], radiographic or bacteriologic screening has typically been used to exclude individuals with subclinical TB before initiating TPT. Therefore, we used such studies (after excluding estimates of the incidence attributable to nonadherence, reinfection, or preexisting drug resistance) to estimate the efficacy of TPT, and the risks of acquiring resistance when it was ineffective, among latent progressors (Additional file 1: Supplemental methods 2b). We assumed that 4R was at least as effective as 6 months of isoniazid and no more effective than 9 months of isoniazid [8]. The risk of acquiring resistance to the TPT drug was assumed to be lower for 4R than for 6H and independent of susceptibilities to non-prescribed drug classes (Additional file 1: Supplemental methods 2d).

We assumed that TPT could also prevent progression from subclinical to active TB, but that it had lower efficacy against subclinical disease than against latent infection (failing to prevent 9% [95%CI 3–18%] of progressions from the subclinical stage that could have been prevented at the latent stage; Table 1). In addition, those who progressed from subclinical to active disease despite TPT were at elevated risk (compared to latent progressors) of acquiring new drug resistance to the TPT drug in the process. For these subclinical progressors who received TPT, efficacy and resistance acquisition were estimated from historical clinical trials in which isoniazid or rifampicin was the only effective drug in a treatment regimen (Additional file 1: Supplemental Methods 2c and 2e).

#### Simulation of outcomes

For each cohort, we simulated TPT in combination with screening for subclinical TB and, alternatively, with only symptom-based screening. We modeled either 4R or 6H as the TPT regimen, comparing each to a reference of no TPT and, secondarily, to one another. The reduction in TPT access associated with a subclinical TB screening requirement was arbitrarily set at 20% for the primary analysis and varied in sensitivity analyses; this value is expected to be setting-specific.

For each combination of regimen, setting, and screening strategy, the total number of active TB cases prevented and the net change in isoniazid and/or rifampicin-resistant TB cases were measured per 1000 individuals screened for TPT eligibility. The active TB cases that we tallied included those resulting from the progression of latent infection or subclinical disease, as well as those resulting from failure or relapse after TB treatment in the future. Drug-resistant TB cases included those with either newly acquired or pre-existing drug resistance; thus, we accounted for the potential of TPT to create new drug resistance, prevent progression of existing drug resistance, and avert the need for future TB treatment courses that could result in the acquisition of drug resistance.

To represent parameter uncertainty, values were drawn simultaneously from beta (if bounded by 0 and 1) or gamma probability distributions (Additional file 1: Tables S1-S5). Results are presented as a median and 95% inner quantile uncertainty range (95%UR) across 5000 independent draws of all parameter values.

We performed probabilistic one-way sensitivity analyses for each parameter by comparing, among all 5000 probabilistically sampled models, the 500 models with the highest values of that parameter to the 500 models with the lowest value of that parameter (while other parameters varied probabilistically as before). We also explored tradeoffs between screening and access, across a range of values for the impact of a screening requirement on TPT access.

## Results

### Outcomes without TPT intervention

**Fig. 2:**
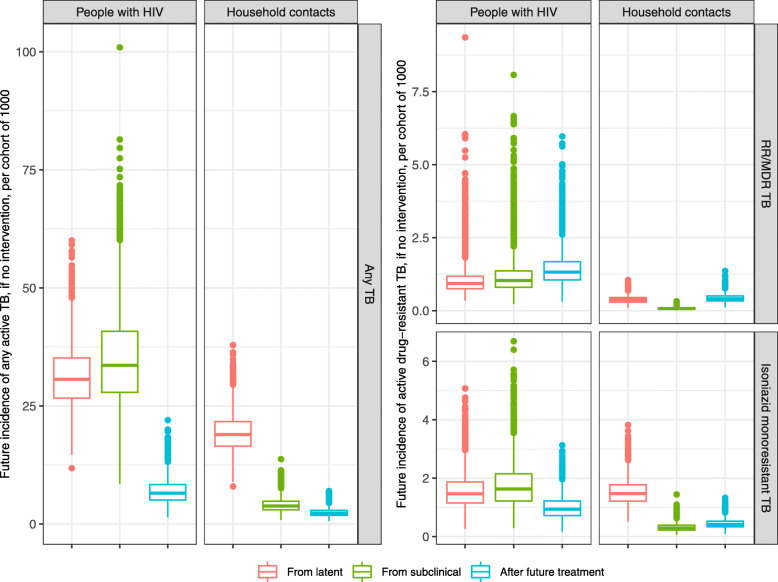
Projected TB cases, in the absence of preventive therapy, among 1000 people newly diagnosed with HIV and 1000 TB household contacts. Simulated outcomes include the incidence of active TB (those cases arising from infections present before the start of the model) and the incidence of isoniazid- and/or rifampicin-resistant TB. Cases are classified based on whether they progressed from TB that was latent or subclinical at the time of TPT consideration (the start of the model) or were a recurrence of TB after non-curative treatment. Boxes show the median and interquartile range of projections when parameters are sampled probabilistically, and dots show outlier simulations that differ from the median by more than 1.5x the interquartile range in either direction

Figure 2 shows the simulated incidence of active TB in each cohort, in the absence of TPT. Among 1000 PWH, our simulation predicted 71 (95%UR 44–111) cases of active TB, of which 43% (34–53%) arose from the progression of latent infection, 48% (38–56%) began as subclinical TB disease at the start of the model, and 9% (5–14%) were recurrences after unsuccessful treatment of one of these active TB cases arising from the latent or subclinical progressor states. The HHC cohort had a lower active TB incidence of 25 (16–37-) per 1000, and a lower proportion of those cases (15% [95%UR 9–34%]) arose from TB disease that was subclinical at the start of the model (Fig. 2).

Focusing on drug resistance, we projected 3.3 (1.8–8.1) future RR/MDR cases and 4.1 (2.0–7.9) isoniazid monoresistant cases among 1000 PWH and 0.9 (95%UR 0.5-1.5) future RR/MDR cases and 2.2 (1.2-3.7) isoniazid monoresistant cases among 1000 HHCs. Nearly 50% of these projected RR/MDR-TB cases and nearly 25% of projected isoniazid monoresistant cases were recurrences after a future course of active TB treatment, either because resistance was acquired during treatment, or because pre-existing resistance made treatment non-curative (Fig. 2).

### Impact of TPT with symptom-only TB screening

**Fig. 3:**
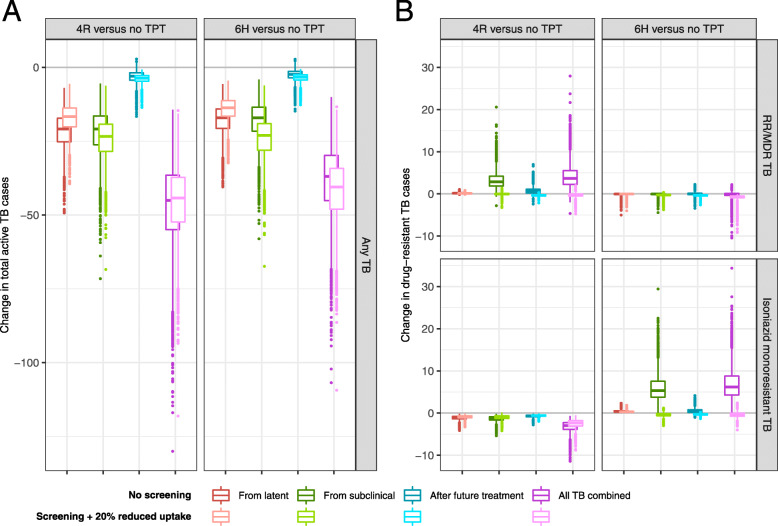
TB incidence after of rifampicin (4R) or isoniazid (6H) preventive therapy, relative to no preventive therapy, in a cohort of 1000 newly diagnosed people with HIV. Outcomes include isoniazid (INH) monoresistant, rifampicin- or multidrug-resistant (RR/MDR), and total incident active TB cases, including relapses/failures after a single round of TB treatment if received. The darker shade of each color shows outcomes with no screening for subclinical TB, such that those with subclinical TB receive single-drug TPT. For comparison, the lighter shade of each color shows outcomes when subclinical TB must be ruled out before TPT; in this figure, it is assumed that this testing requirement reduces access to preventive therapy by 20%. Boxes show a median and interquartile range of projections when parameters are sampled probabilistically, and dots show outlier simulations that differ from the median by more than 1.5x the interquartile range in either direction. Analogous results for a cohort of household contacts are shown in Additional file 1: Figure S1, and for only under-age-5 contacts in Additional file 1: Figure S5

When only symptom-based screening for TB disease was performed (i.e., when those with subclinical TB received TPT along with the latently infected), and 4R was used as the TPT regimen, 45 (95%UR 24-79) active TB cases were averted among 1000 PWH; these represented 64% (95%UR 40-89%) of all incident active TB cases not attributable to future TB exposure (Fig. 3). Similarly, among 1000 HHCs, symptom screening and 4R prevented 17 (95%UR 9–29) active TB cases or 68% (95%UR 43–94%) of the incident cases arising from infections present at the time of the intervention (Additional file 1: Figure S1). Due to the lower assumed efficacy of 6H and a higher prevalence of preexisting resistance to isoniazid versus rifampicin, using 6H as the TPT regimen after symptom screening averted 37 TB cases (19–66, 52–73%) among PWH and 13 TB cases (7–23, 34–75%) among HHCs (Fig. 3 and Additional file 1: Figure S1).

Meanwhile, 4R also averted 3.0 (95% UR1.3–6.1) isoniazid-monoresistant cases among PWH, while adding 3.5 (0.2–11.0) rifampicin-resistant cases; most (82%, 95%UR 72–97%) of the net increase in rifampicin resistance was rifampicin-monoresistant as opposed to multidrug-resistant TB. In the HHC cohort, where overall TB incidence was lower, these projections decreased to 1.6 (0.8–2.9) isoniazid monoresistant cases averted and 0.4 (0–1.5) RR/MDR cases (86% [73–99%] rifampicin monoresistant) added per 1000 individuals. Using 6H as the TPT regimen, by contrast, increased net isoniazid resistance (adding 6.2 [1.8–16] isoniazid-resistant cases among PWH and 0.8 [0.2–2.3] among HHCs), while having minimal impact on net RR/MDR TB (median 0.1 case averted [95%UR 2.8 averted to 0.7 added] and median 0.1 case averted [95%UR 0.2 averted to 0.1 added], respectively).

Thus, compared to no TPT, each TPT regimen was expected to prevent more than 10 cases of active TB for each added case of resistance to that TPT drug. Specifically, 4R prevented median 12 (95%UR 3–102) active TB cases among PWH and 37 (95%UR 9–580) among HHCs for each rifampicin-resistant (monoresistant or MDR) case added (Table 3). Similarly, 6H prevented 6 [IQR 2–26] active TB cases among PWH and 15 (95%UR 5–98) among HHCs for each isoniazid-resistant (monoresistant or MDR) case added. If we compared 4R and 6H head-to-head with only symptom-based screening, the choice to use 4R rather than 6H led to reductions in incident active TB (uncertain in magnitude, and largely attributable to our assumption that 4R is somewhat more efficacious), and it traded modest a net increase in isoniazid resistance for a smaller net increase in rifampicin resistance: among PWH, the decision to use 4R instead of 6H added one case of rifampicin resistance for every 2 (0.4–14) incremental cases of active TB and every 2 (1–8) cases of isoniazid monoresistance averted, compared to 6H. Among the HHC cohort (which had fewer subclinical progressors and a higher prevalence of isoniazid monoresistance), the analogous estimates were one case of rifampicin resistance added for every 7 (2–80) active TB cases averted and every 5 (2–40) isoniazid-resistant cases averted.

**Table 3:** Median outcomes of TPT with 4R in cohorts of 1000 PWH or 1000 TB household contacts, compared to no TPT or to 6H, under different scenarios of subclinical TB screening and associated intervention access

	4R, symptom screening only	4R, Subclinical TB screening, same access	4R, Subclinical TB screening, 20% reduced access	6H, symptom screening only	6H, Subclinical TB screening, same access	6H, Subclinical TB screening, 20% reduced access
**PWH cohort**						
Expected cases of active TB without TPT	71	71	71	71	71	71
Expected cases of active TB with TPT #	25	16	27	33	20	31
Net change in total active TB cases (versus no TPT) ^a^	−45	−55	−44	−37	−51	−41
Net change in INH monoresistant cases (versus no TPT)	−3	−2.9	− 2.3	6.2	−0.48	− 0.38
Net change in RIF monoresistant cases (versus no TPT)	3	0.3	0.24	−0.23	−0.11	− 0.09
Net change in MDR cases (versus no TPT)	0.63	−0.56	−0.45	0.18	−0.59	− 0.47
Net change in total DR cases (versus no TPT)	−0.85	−1.5	− 1.2	1.1	− 0.69	− 0.55
Symptomatic TB cases averted per RR/MDR added (vs no TPT)	12	^b^	^b^	^b^	^b^	^b^
INH monoresistance averted per RR/MDR added (vs no TPT)	0.78	^b^	^b^	^b^	^b^	^b^
Symptomatic TB cases averted per RR/MDR added (vs 6H)	2	7.2	7.2	NA	NA	NA
INH monoresistance averted per RR/MDR added (vs 6H)	2.3	4	4	NA	NA	NA
**Household contact cohort**						
Expected cases of active TB without TPT	25	25	25	25	25	25
Expected cases of active TB with TPT #	7.8	6.8	10	12	10	13
Net change in total active TB cases (versus no TPT) ^a^	−17	−18	−15	−13	−15	−12
Net change in INH monoresistant cases (versus no TPT)	−1.6	− 1.6	−1.3	0.84	−0.02	−0.02
Net change in RIF monoresistant cases (versus no TPT)	0.39	0.09	0.07	−0.06	−0.05	− 0.04
Net change in MDR cases (versus no TPT)	0.02	−0.1	−0.08	0	−0.06	− 0.05
Net change in total DR cases (versus no TPT)	−0.56	− 0.56	−0.44	0.11	−0.09	− 0.07
Symptomatic TB cases averted per RR/MDR added (vs no TPT)	37	^b^	^b^	^b^	^b^	^b^
INH monoresistance averted per RR/MDR added (vs no TPT)	3.5	^b^	^b^	^b^	^b^	^b^
Symptomatic TB cases averted per RR/MDR added (vs 6H)	7.2	24	24	NA	NA	NA
INH monoresistance averted per RR/MDR added (vs 6H)	5	13	13	NA	NA	NA

^a^Active TB that develops from subclinical or latent TB that was present at enrollment

^b^No net increase in RR/MDR TB in > 90% of simulations

Abbreviations: *CXR* chest radiogram, *TPT* tuberculosis preventive treatment, *TB* tuberculosis, *PWH* patients newly diagnosed with HIV, *INH* isoniazid, *RIF* rifampicin, *RR* rifampicin monoresistant, *MDR* multidrug-resistant, *DR* drug-resistant, *4R* rifampicin, 4-month regimen, *6H* isoniazid, 6-month regimen

### Impact of TPT after subclinical TB screening

The lighter-colored boxes in Fig. 3 and Additional file 1: Figures S1 and S2 show the impact of 4R or 6H when cohorts were screened for subclinical TB prior to initiating TPT, under the arbitrary assumption that the screening requirement reduced access to TPT by 20%. The ability of TPT to prevent progression of latent TB was reduced in proportion to this reduction in access (Fig. 3A and Additional file 1: Figure S1 Panel A, red boxes), but that the effect was offset by the benefits of appropriately treating subclinical TB that would have progressed to active disease had it been treated with only TPT (green boxes), resulting in similar overall active TB incidence (pink boxes; with 4R preventing 62% [51–73%] of incident active TB among PWH and 58% [41–76%] among HHCs, for example).

**Fig. 4:**
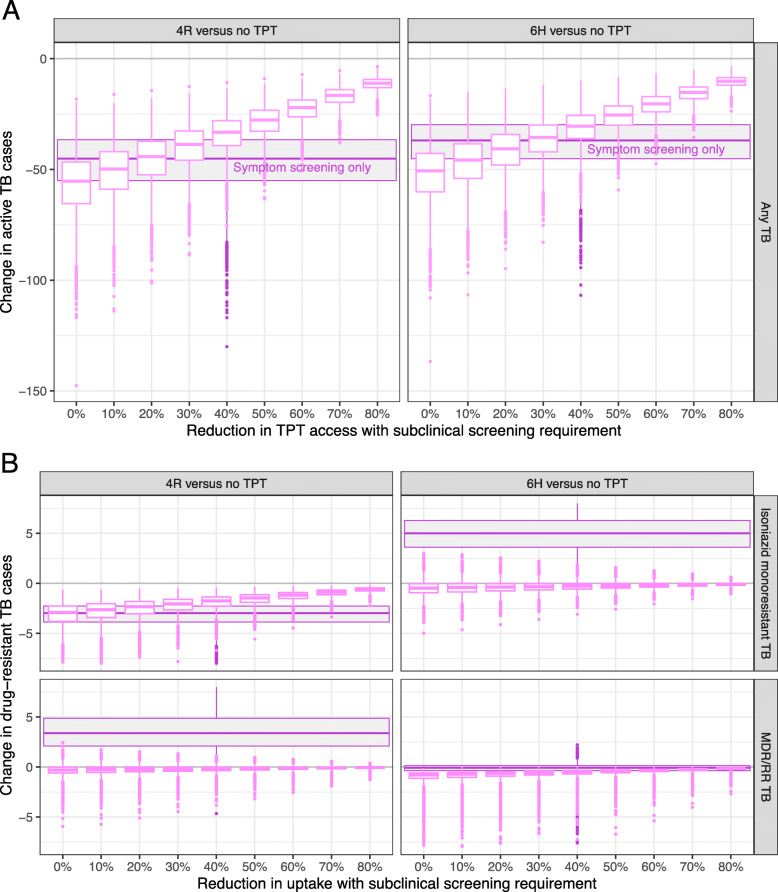
Effects of subclinical TB screening on TB incidence after TPT, for various magnitudes of effect on TPT access, among PWH cohort. Lighter-colored boxes show the median projection and interquartile range for each TB incidence outcome at various reductions in access, overlaid on a darker box showing the median projection and interquartile range for the impact of TPT with symptom-only screening (and no reduction in access). Results are shown for a cohort of 1000 household contacts; analogous results for a cohort of household contacts are available in Additional file 1: Figure S3

If the screening requirement reduced TPT access by a larger amount, then its effect on overall TB incidence was detrimental; for example, among 1000 PWH, if screening for subclinical TB reduced intervention access by 50%, then it lowered the impact of 4R to 28 (17–45) cases of active TB averted, compared to 45 (24–80) averted by 4R when paired with symptom-only screening. The predicted reduction in access at which a screening requirement had no net effect on the overall incidence of active TB ranged from 10 to 30% depending on the cohort and regimen, with the lower thresholds estimated for the more efficacious regimen (4R) (Fig. 4 and Additional file 1: Figure S3).

Although screening for subclinical TB could reduce the overall TB prevention impact of TPT if it significantly restricted access, it had the benefit of eliminating net acquisition of drug resistance, because the prevention of TB cases that would have acquired resistance during future treatment outweighed the number of individuals who acquired resistance during TPT in most simulations (Fig. 3B, light-colored boxes).

### Sensitivity analysis

In one-way sensitivity analysis, the largest contributors to uncertainty about the absolute number of TB or drug-resistant cases prevented by TPT were the estimated efficacy of the TPT regimens and the proportion of the cohort expected to progress from latent or subclinical to active TB. Because we did not differentiate TPT efficacy between PWH and HHCs, the different prevalence of the subclinical progressor state and different future cumulative incidence of latent TB progression were the main sources of difference between the PHW and HHC cohorts. Drug-resistance-related outcomes were also sensitive to the estimated risk of resistance acquisition when subclinical TB was not cured by TPT (Additional file 1: Figure S3).

## Discussion

In this TB preventive therapy modeling study, a 4R TPT regimen—when paired with sensitive screening for subclinical TB and available to at least 80% of the eligible population—was expected to prevent > 60% (and highly likely to prevent > 40%) of progression to active TB among both PWH and household contacts of TB patients, while also reducing isoniazid-resistant TB and having no clinically meaningful net effect on rifampicin-resistant TB. Thus, in settings where testing for subclinical TB is available with only a small reduction in TPT access, such testing has the advantage of allowing patients to benefit from the short duration, efficacy, and tolerability [8] of the 4R regimen without risk of adding to the burden of rifampicin resistance. X-ray or other tests for subclinical TB may not be readily available in high-TB-burden settings, however. Despite improving radiographic technology and illustrative successes in implementing it for TB screening in some low-resource settings [79–81], requirements to perform sensitive screening may limit access to TPT in many parts of the world. In clinical situations where screening for subclinical TB can be offered, it has both TB prevention and drug resistance prevention advantages, and our results can aid in economic evaluations of the cost-effectiveness of such screening. If, however, subclinical TB screening will substantially reduce TPT access in certain patient populations or care settings, then guidelines that support initiation of TPT after a negative symptom screen are likely to maximize TB prevention. For the 4R regimen, we estimated a 10–20% reduction in access as the threshold above which a symptom-only screening policy would prevent more active TB (while also minimizing drug resistance) than implementing TPT with a subclinical TB screening requirement. The precise threshold at which preventive efficacy offsets reductions in access is uncertain; however, our results suggest that it may range from 0 to 60% depending on the prevalence and outcomes of subclinical TB in the TPT eligible population.

When considering allowing symptom-only screening to maximize TPT access, decision-makers must weigh maximal prevention of TB (achievable with the 4R regimen and broad eligibility criteria) against the potential selection of rifampicin and/or isoniazid resistance. Our analysis indicates that without subclinical TB screening, 4R would avert multiple cases of active TB for every case of rifampicin resistance added (point estimates 12 among PWH and 37 among HHCs, though with wide uncertainty); this corresponded to an absolute risk of one case of rifampicin resistance for every 300 (among PWH) to 2400 (among HHCs) recipients of the 4R TPT regimen. This risk may be judged to be acceptable; if rifampicin-susceptibility testing (e.g., Xpert MTB/RIF) and alternative regimens for rifampicin-monoresistant TB are available to people with a known rifampicin TPT history, then the rifampicin resistance that develops from inadvertent 4R monotherapy may be readily detected and appropriately treated. Alternatively, if subclinical TB screening is not feasible, but a small increase in the risk of rifampicin resistance is considered unacceptable because of rifampicin’s crucial role in active TB treatment regimens, then decision-makers may forego the efficacy, safety, and operational advantages of the 4R regimen in lieu of the 6H regimen (which we estimated would add more isoniazid-resistant cases than the rifampicin resistance added by 4R, and would prevent slightly less TB overall, but would avoid rifampicin resistance).

Among models of TPT and drug resistance, our analysis is the first to our knowledge to model rifamycin-based regimens and one of few that explicitly simulate the prevalence and outcomes of subclinical TB. However, our work builds on previous models of the interplay of TPT’s clinical benefits and potential drug-resistance risks [50, 82]. Our results are consistent with a model of multidrug-resistant TB preventive therapy and acquired fluoroquinolone resistance [83], in the conclusion that among people with latent TB, resistance generated by TPT is likely to be offset by a reduction in future opportunities for resistance to be acquired during TB treatment.

Because clinical trials of TPT identify and exclude those with subclinical TB, there is considerable uncertainty about the outcomes of TPT in this population. Many have paucibacillary disease that may be curable with a single TPT drug; however, even high bacillary burden (e.g., smear-positive) prevalent TB is often asymptomatic [51]. Although our model provides a high level of certainty for some findings—for instance, that 4R paired with subclinical TB screening will not lead to large increases in the prevalence of rifampicin resistance—other results are subject to uncertainties about the course of subclinical TB. For example, the number of active TB cases preventable by each TPT regimen depends not only on the efficacy of each TPT regimen against latent TB, but also on how often subclinical TB progresses to symptomatic disease and how often TPT, if given at the subclinical stage, is able to halt that progression. To allow more precise estimates, data are needed on outcomes after TPT in patient populations from which subclinical TB has not been systematically excluded. In the ongoing global scale-up of TPT without a strict requirement for chest radiography, programmatic data on the subsequent incidence of TB and burden of drug resistance should be monitored to better understand these outcomes.

Our uncertainty ranges reflect uncertainty in the outcomes of monotherapy for progressive subclinical TB, including the probabilities of cure and the risks of resistance acquisition. Available data reflect outcomes of single-drug therapy for truly latent infection and for symptomatic active disease, but the spectrum of bacillary burden in subclinical disease and the associated treatment outcomes are less well characterized. We also modeled only single-drug TPT regimens. Our results may be interpreted as an upper bound for the resistance risks associated with 2-drug regimens such as 3HP and 3HR, but further work is needed to understand the extent to which outcomes differ for a two-drug TPT regimen, particularly when dosed at a long interval relative to isoniazid’s half-life. We simplified drug resistance as dichotomous and assumed that a given TPT regimen was equally efficacious in patients with and without HIV. HIV may theoretically reduce the ability of single-drug therapy to cure subclinical disease, but data from active TB treatment among PWH on antiretroviral therapy [84] and from clinical trials of 4R TPT [85] suggest that differences are minimal. Because we did not use a transmission model, our analysis does not consider how the evolution of TB epidemics affects TPT-associated drug resistance. Finally, we focused only on TB outcomes within the modeled cohort and did not consider other outcomes that may differ in important ways between TPT strategies. For example, screening for subclinical TB will incur the costs of radiographic screening and confirmatory testing, with cost-effectiveness implications. It may also lead to adverse events from treating patients for subclinical TB, including some who would never have developed symptoms; these adverse effects could be limited by a test that was specific for those subclinical cases that are progressive, but no such test currently exists. On the other hand, we did not account for the potential that identifying and treating patients with subclinical TB may, in some cases, prevent asymptomatic transmission, including transmission from those whose disease might eventually resolve without intervention.

## Conclusions

In summary, because subclinical TB is often present in populations considered for TPT, chest radiography is advisable both to increase TB detection and to minimize the risk that TPT could generate drug resistance in people with active TB. Such testing is likely to reduce TB incidence even if the requirement for testing reduces TPT access by as much as 20%. With testing for subclinical TB in place, 4R is predicted to have better TB outcomes than 6H without posing resistance-related risks. When such screening is infeasible and active TB must be excluded based on symptoms alone, regimen selection will need to account for the importance of incident TB and isoniazid resistance relative to rifampicin resistance, while also considering the tolerability and cost-effectiveness advantages of 4R.

## Supplementary information

**Additional file 1: Supplemental methods, Tables S1-S6, and Figures S1-S5.** Supplemental methods – Additional details of primary human subjects data collection, estimation of model parameters, state-transition model, and approach to probabilistic parameter sampling. **Table S1.** Parameter estimates, sizes of latent and subclinical progressor populations. **Table S2.** Parameter estimates, preventive therapy efficacy and related parameters. **Table S3.** Parameter estimates, preventive therapy resistance acquisition. **Table S4.** Parameter estimates, TB treatment outcomes. **Table S5.** Composition of cohorts with respect to initial drug resistance and age or CD4 count. **Table S6.** Correspondence between baseline culture-positive TB prevalence and 3-month clinical TB incidence in cohort of people with newly diagnosed HIV and a negative TB symptom screen in Kwa-Zulu Natal. **Fig S1.** – Projected outcomes of 4R or 6H, each compared to no TPT, among a cohort of 1000 all-age Household contacts. **Fig S2.** Head-to-head comparison of TB outcomes after TPT, comparing 4R regimen to 6H. **Fig S3.** Effect of reduced access on the impact of subclinical TB screening prior to TPT, among HHC cohort. **Fig S4.** Sensitivity of key results to individual parameters. Fig S5 – Projected outcomes of 4R or 6H, each compared to no TPT, among 1000 household contacts, when TPT is only considered for the 17% of contacts who are under age 5

## Data Availability

Code and summary data used for this analysis are available at https://github.com/eakendall/RifTPT.

## Abbreviations

TB: Tuberculosis
TPT: Tuberculosis preventive therapy
6H: Isoniazid for 6 months
4R: Rifampicin for 4 months
3HP: Weekly rifapentine and isoniazid for 3 months
PWH: People living with HIV
HHCs: Household contacts
95%UR: 95% uncertainty range
