# Supplementary material for: Isoniazid or rifampicin preventive therapy with and without screening for subclinical TB: a modeling analysis
Source: BMC Med. 2021 Dec 14;19:315. doi: 10.1186/s12916-021-02189-w (PMC8670249; doi:10.1186/s12916-021-02189-w)

**Additional file 1**

**Isoniazid or rifampicin preventive therapy with and without screening for subclinical tuberculosis: A modeling analysis**

Kendall et al.

Table of Contents

[SUPPLEMENTAL METHODS 2](#_Toc87523656)

[1. Human subjects data collection: 2](#_Toc87523657)

[a. HIV cohort enrollment 2](#_Toc87523658)

[b. Household contact cohort enrollment 2](#_Toc87523659)

[2. Details of parameter estimation 3](#_Toc87523660)

[a. Prevalence of “subclinical progressor” and “latent progressor” states 3](#_Toc87523661)

[2.a.1) Cohort of PWH: 3](#_Toc87523662)

[2.a.2) Household contacts 4](#_Toc87523663)

[Table S1: Parameter estimates, sizes of latent and subclinical progressor populations 5](#_Toc87523664)

[b. Efficacy of preventive therapy among latent progressors 5](#_Toc87523665)

[c. Efficacy of preventive therapy against progressive subclinical TB 6](#_Toc87523666)

[Table S2: Parameter estimates, preventive therapy efficacy and related parameters 7](#_Toc87523667)

[d. Risk of acquiring resistance when latent, regimen-susceptible TB progresses despite preventive therapy 7](#_Toc87523668)

[e. Risk of acquiring resistance when subclinical, initially-regimen-susceptible TB progresses despite preventive therapy 9](#_Toc87523669)

[Table S3: Parameter estimates, preventive therapy resistance acquisition 9](#_Toc87523670)

[f. Risks of failure or relapse after TB treatment, with or without acquired drug resistance, by initial resistance phenotype 10](#_Toc87523671)

[Table S4: Parameter estimates, TB treatment outcomes 10](#_Toc87523672)

[g. Prevalence of drug resistance: 11](#_Toc87523673)

[h. Relative sizes of cohort strata 11](#_Toc87523674)

[Table S5: Composition of cohorts with respect to initial drug resistance and age or CD4 count 11](#_Toc87523675)

[i. Sensitivity, access, and requirements of subclinical TB screening 11](#_Toc87523676)

[3. State transition steps: 12](#_Toc87523677)

[4. Probabilistic parameter sampling: 12](#_Toc87523678)

[SUPPLEMENTAL RESULTS 13](#_Toc87523679)

[Table S6. Correspondence between baseline culture-positive TB prevalence and 3-month clinical TB incidence in cohort of people with newly diagnosed HIV and a negative TB symptom screen in Kwa-Zulu Natal. 13](#_Toc87523680)

[Figure S1: Projected outcomes of 4R or 6H, each compared to no TPT, among a cohort of 1000 all-age Household contacts. 14](#_Toc87523681)

[Figure S2: Head-to-head comparison of TB outcomes after TPT, comparing 4R regimen to 6H 15](#_Toc87523682)

[Figure S3: Effect of reduced access on the impact of subclinical TB screening prior to TPT, among HHC cohort 16](#_Toc87523683)

[Figure S5: Projected outcomes of 4R or 6H, each compared to no TPT, among 1000 household contacts, when TPT is only considered for the 17% of contacts who are under age 5 18](#_Toc87523684)

SUPPLEMENTAL METHODS

1. Human subjects data collection:
2. HIV cohort enrollment

In brief, we enrolled adults who presented for voluntary HIV testing at the iThembalabantu Clinic in the Umlazi township of KwaZulu-Natal, South Africa from September 2013 to February 2019. The clinic provides free clinic- and community-based HIV care and treatment for over 10,000 PLHIV. We enrolled English or Zulu speaking adults ≥18 years of age, who were not pregnant, did not report previous ART use, and had not taken anti-fungal therapy in the preceding three months. The study was approved by the University of Washington’s Institutional Review Board (IRB #49563) and the University of KwaZulu-Natal’s Medical Research Ethics Committee (Protocol #BF052/13). All study participants provided written informed consent.

At the enrollment visit, we collected data using a sociodemographic questionnaire and HIV counselors performed serial rapid HIV testing according to South African guidelines.[33] Among PLHIV participants, research nurses obtained a medical history, administered a clinical symptom questionnaire, and ordered blood and urine samples for clinic- and laboratory-based testing, including CD4 count. All participants received routine medical care, according to local guidelines. Research assistants contacted participants by phone and reviewed medical charts at three, six and 12-months post-enrollment to complete a clinical questionnaire and assess their treatment outcomes.

All participants were followed for 12 months. We reviewed each participant’s medical chart, including hospitalization, and attempted at least three calls to participants. Any hospitalized participant had an additional hospital chart review to determine the cause of hospitalization. For those whose vital status could not be obtained at the study end, we searched the South African national death registry. At the study end, all participants were categorized as either retained in care at the study clinic, transferred to another HIV clinic, lost to clinical follow-up, or deceased.

1. Household contact cohort enrollment

Household contacts were evaluated as part of programmatic implementation research initiative to increase tuberculosis diagnosis and prevention in Pakistan. All patients with drug-susceptible TB were counseled to bring their families for contact evaluation, and those who did not come received a reminder phone call followed by an attempted home visit. The operational research study was approved by Interactive Research and Development’s Institutional Review Board (#IRD_IRB_2016_08_003) and the National Bioethics Committee of Pakistan (#NBC-270).

All patients were verbally screened by a health worker for symptoms of cough for 2 weeks or more, fever, or night sweats. All contacts (regardless of symptoms) were also offered a clinical evaluation by a medical doctor and CAD4 chest radiography. Every attempt was made to get a sputum specimen for testing with Xpert MTB/RIF from all contacts. At the recommendation of the evaluating physician, some contacts underwent additional testing including gastric aspirate, fine needle aspirate cytology, biopsy, ultrasound, CT scan, or culture.

1. Details of parameter estimation
2. Prevalence of “subclinical progressor” and “latent progressor” states

For both cohorts, our modeling approach focused on future active TB cases arising from infections that were present at “time 0” when evaluation for TPT occurred. Furthermore, we did not specify the timing of those future progressions to active TB, but only that they occurred at time >0, arose from infections present at time 0, and therefore were potentially affected by TPT administered at time 0.

2.a.1) Cohort of PWH:

For the cohort of PWH, we had 12 months of clinical data on active TB incidence, stratified by baseline CD4 count. We classified individuals with incident active TB as having had progressive subclinical TB at baseline if they were asymptomatic at baseline but diagnosed with TB within 3 months of ART initiation. Consistency between this proportion, and the prevalence of sputum culture positivity at enrollment, was verified in a sub-study that performed culture at enrollment on a random subset of the HIV patient cohort, as shown in table S6.

We then estimated the lifetime incidence of progression from latent TB, by (a) estimating what proportion of incidence in months 4-12 arose from infections present at time 0, and then (b) extrapolating the lifetime incidence of progression from the rate in months 4-12. These estimates incorportated the following assumptions:

- - - 1. For people with a baseline CD4 >350, being on ART for >1 year lowers the TB incidence rate by a factor of 0.72 [34].
      2. Beyond the first year on ART, the risk of TB reactivation among PWH is independent of the initial CD4 count (consistent with [35]).
      3. For HIV-uninfected people who are enrolled at some arbitrary time, and who are equally likely to have been infected with TB at any point in the past, approximately 30% of all future progressions to active TB (from infections present at enrollment) will occur in the first year of follow up.

This timing of reactivation with HIV-uninfected individuals was estimated from data from Wallgren [36] (as summarized by Behr and colleagues [37]), using r(t) to represent the proportion of reactivations that occur by t years after infection (e.g. r(1 year) = 54%, r(2 years) = 72%, r(3 years) = 81%. For an infection that occurred T years before enrollment, the probability of reactivation in the one year after enrollment is c*(r(T+1) – r(T)), where c is the lifetime cumulative incidence of reactivation, and the probability of reactivation at any time after enrollment is c(1-r(T)). Numerically integrating, first assuming an average of N=30 years of possible exposure before enrollment, we estimate the proportion of future reactivations that will occur in the next year as$\int_{T=0}^{N} r\left( T+1 \right)-r\left( T \right)dT$ / $\int_{T=0}^{N} 1-r\left( T \right)dT$ = 31%. This estimate increases to 34% if N=20, and decreases to 26% if N=50.

- - - 1. HIV compresses the timeline of progression to active TB after infection, reducing the proportion of reactivation that occurs beyond one year from approximately 70% to approximately 56%.

This was estimated using the same approach described above, after accounting for the association between untreated HIV and increased odds that an incident case resulted from recent transmission (estimated odds ratio 1.26, [38]). If HIV compresses the reactivation timeline by a factor *h*, then this association corresponds to r(hy)/(1- r(hy)) = 1.26 r(y)/(1-r(y)), where y is the recent time period. Solving for each of multiple possible values of y, h=1.33 if recent transmission is defined as y=1 year, h=1.25 for y = 2 years, h=1.24 for y = 3 years, h=1.14 for y = 4 years, and h =1.39 for y = 5 years. These estimates are all similar, and we use the average of h = 1.27 as our point estimate. Therefore, for PWH not on ART, the proportion of reactivation expected to occur within 1 year after enrollment is (solving as above) $\int_{T=0}^{N} r\left( h(T+1) \right)-r\left( hT \right)dT$ / $\int_{T=0}^{N} 1-r\left( hT \right)dT$ . This is equal to 36% for N=20 years of potential exposure (30% if N=50 years, 40% if N=20 years). Accounting for an ART-associated reduction in the incidence rate beyond the first year (relative risk 0.72, above), the proportion of future reactivation incidence occurring beyond 1 year in an ART cohort would be approximately 0.72*(1-0.36)/(0.36 + 0.72*(1-0.36)) = 56%. In other words, the cumulative future reactivation incidence beyond 1 year would be 0.56/(1-0.56) = **1.27** times the reactivation incidence in the first year.

Thus, for each baseline CD4 stratum *s*, we estimated the incidence of TB progression within the first year as I_s6_/N_s6_ + I_s12_/N_s12_ , where N_s6_ and N_s12_ are the number of individuals followed to 6 or 12 months, respectively, and I_s6_ and I_12s_ are the number diagnosed with incident TB between months 3-6 and months 6-12, respectively. Beyond the first year, applying the estimate of 1.27 above, we estimated the cumulative progression incidence for all strata as 1.27 *( $\sum_{s} I_{6s}/ \sum_{s} N_{6s}$+ $\sum_{s} I_{12s}/ \sum_{s} N_{12s})$.

Uncertainty in these estimates was modeled as uncertainty in the ratio of subclinical progressors to latent progressors in each sub-cohort.

2.a.2) Household contacts

For the cohort of household contacts, we used

1. baseline data from the Pakistan cohort to estimate the prevalence of active and subclinical disease at the time of contact investigation,
2. systematic review data [39, 40] to estimate the ratio of TB disease prevalence (active plus subclinical, at the time of initial contact investigation) to future reactivation incidence,
3. historical data on outcomes of untreated TB to estimate what proportion of subclinical TB would resolve rather than progressing to active TB, even in absence of any intervention.

The prevalence of active and subclinical TB identified in the Pakistan cohort, by age stratum, are shown in Table 2 in the main text.

For adults, we estimated the ratio of prevalent to incident TB from Fox et al [40]. In this review, 4.5% of household contacts in low and middle income countries developed active TB within 5 years; we assumed that the unobserved reactivation incidence beyond 5 years was offset by observed incidence that had not arisen from infections present at enrollment (from rather from new infections that occurred and progressed during 5-years of follow up); thus we assumed that this overall 5-year incidence was a reasonable approximation of the lifetime reactivation incidence arising from infections present at enrollment. Comparing this 5-year cumulative incidence of 4.5% to the baseline prevalence of 3.1% in the same systematic review, we estimated a ratio of 1.45 future progressions to incident active TB per prevalent case at enrollment, among adult contacts. In the recent Pakistan cohort, the baseline prevalence of TB was lower, but we assumed that the same ratio of prevalence to incidence would apply. Thus, we multiplied the baseline prevalence of TB by this multiplier to estimate the prevalence of the latent progressor state among adults (Table S1).

For children, we calculated a similar ratio using data from Martinez et al [39]: we used diagnoses within 90 days to estimate baseline prevalence, and a 2-year follow up window among children who did not receive TPT (any TST status, excluding baseline prevalent cases and stratifying by age <5 or ≥5 years) to estimate cumulative future reactivation incidence. The resulting estimates were consistent with estimates calculated separately from data reviewed by Marais and colleagues [41]. The ratio of prevalence to incidence was applied to the baseline prevalence in the Pakistan cohort to estimate the number of latent progressors (Table S1).

To estimate the number of subclinical progressors, we took the prevalence of asymptomatic baseline disease in each age stratum of HHCs, and multiplied it by an estimate that 50% (30-70%) of subclinical disease would progress to active disease if untreated [42].

The ratio between this subclinical progressor prevalence and the latent progressor prevalence (Table S1) was used as an input to the TPT model.

In order to capture correlation between these estimated quantities for different strata and settings, uncertainty was modeled both at the level of individual strata (in the prevalence of latent progressors and the ratio of subclinical progressors to latent progessors) and as a multiplier that was applied to estimates of the same quantity for all strata and for both settings.

Table S1: Parameter estimates, sizes of latent and subclinical progressor populations

| **Parameter** | **Cohort** | **Stratum (CD4 or age)** | **Mean estimate** | **Standard deviation*** | **Sampled distribution** | **References** |
| --- | --- | --- | --- | --- | --- | --- |
| Proportion of cohort who are latent progressors | Overall multiplier |  | 1 | 0.2 | gamma |  |
| “ | PWH | CD4<100 | 0.0453 | 0.005 | beta | Primary data and [35, 37, 38, 86]; estimation described in section 2.a.1 above |
| “ | PWH | CD4 100-200 | 0.0474 | 0.005 | beta | “ |
| “ | PWH | CD4 200-350 | 0.0282 | 0.003 | beta | “ |
| “ | PWH | CD4 >350 | 0.0239 | 0 | beta | “ |
| “ | HHC | Age <5yo | 0.0061 | 0.001 | beta | Primary data and ^23^[39, 40]; estimation described in section 2.a.2 above |
| “ | HHC | Age 5-15yo | 0.0366 | 0.002 | beta | “ |
| “ | HHC | Age >15yo | 0.0124 | 0 | beta | “ |
| Number of subclinical progressors per latent progressor | Overall multiplier |  | 1 | 0.2 | gamma |  |
| “ | PWH | CD4<100 | 0.93 | 0.1 | gamma | Ratio of subclinical progressors (Table 2) to latent progressors (above) |
| “ | PWH | CD4 100-200 | 0.81 | 0.1 | gamma | “ |
| “ | PWH | CD4 200-350 | 1.27 | 0.15 | gamma | “ |
| “ | PWH | CD4 >350 | 1.33 |  | NA | “ |
| Number of subclinical prevalent cases per latent progressor | HHC | Age <5yo | 1.42 | 0.1 | gamma | “ |
| “ | HHC | Age 5-15yo | 0.37 | 0.05 | gamma | “ |
| “ | HHC | Age >15yo | 0.35 |  | NA | “ |
| Probability of spontaneous resolution of subclinical TB | HHC | Additional multiplier, applied to household contact cohort | 0.5 | 0.1 | beta | [42]; applies to household contact cohort estimates because measured prevalence was all subclinical TB rather than only progressions from subclinical to active TB |

* The desired coefficient of variation was based on binomial confidence intervals from clinical cohorts, inflated to reflect additional uncertainty in the data (e.g. non-bacteriologic diagnoses in children, estimated reactivation incidence beyond year 1). A multiplier with CV=20% was applied to all subgroups, and additional variability was modeled in the estimate for each other subgroups relative to a reference subgroup for each setting.

- 1. Efficacy of preventive therapy among latent progressors

For the 4R TPT regimen, we start with the odds ratios for incident TB under placebo-controlled clinical trial conditions (δ_4R_), as estimated in network metaanalyses [44]. We used an estimated cumulative incidence of TB with placebo (γ) of 5% [45] to convert odds ratios to a relative risk ρ_4R_:

ρ_4R_ = δ_4R_ / (1 - γ+γδ_4R_)].

We then adjust this estimate for estimates of baseline drug resistance, TPT nonadherence, and incidence arising from future infection, in order to estimate the impact of 4R TPT on future reactivation incidence as an adjusted risk ratio ρ’_4R_, defined relative to placebo among patients who have a rifampicin-susceptible infection and take enough TPT that it could place them at risk for acquired resistance if subclinical TB were missed. Specifically, before estimating ρ’_4R_, we removed from both arms of clinical trials the proportion of incidence that was estimated to have occurred as a result of reinfection after TPT (π). We also limited our estimates of δ_4R_ to the prevention of rifamycin-susceptible TB. And, finally we inflated our efficacy estimates based on the estimated proportion of patients (τ) who were susceptible and adherent to the TPT regimen; a treatment that reduces disease risk by an average amount R among the proportion τ who take the treatment and are susceptible to it will reduce risk by an average of *Rτ* across the entire patient population.

We estimated π as a ratio of the TB incidence attributed to recent transmission in medium- to low-burden trial sites (estimated at 10 per 100,000 population per year [32, 46]) to the observed incidence among TPT trial participants who received placebo (~1 per 1000 person years in Menzies et al 2018 4R trial).

We estimated τ as 90%, based on reported adherence in several recent TPT trials [7, 47, 48], assuming that half of those who are lost to follow up may be cured by a partial TPT course [49], and combining this nonadherence with a <2% estimated prevalence of rifampicin resistance among the modeled TPT-eligible populations. Our simulation also accounts for the outcomes of TPT among those with pre-existing resistance and/or treatment nonadherence, but it models them separately.

Therefore, we estimated the 4R efficacy parameter ρ’_4R_ by excluding reinfections from the clinical trial relative risk ρ_4R_, and then adjusting for the proportion of patients *τ* who could benefit:

$$1-{\rho^{'}}_{4R}= \frac{1-\rho_{4R}-\pi}{(1-\pi)\tau}$$

We then parametrized the efficacy of 6H relative to that of 4R, assuming that a proportion α of the TB progressions that could be prevented by 4R would not be prevented by 6H: i.e.,

ρ’_6H_ = ρ’_4R_ + α (1- ρ’_4R_)

The resulting parameter estimates are shown in Table S2 below.

We assumed that 4R and 6H had no effect on rifampicin-resistance and isoniazid-resistant TB, respectively, and we ignored potential effects of TPT on new infections occurring during or after the TPT course [50], apart from accounting for estimated rates of reinfection in the incidence rate ratios observed in clinical trials of TPT.

Because of how we defined the latent progressor and subclinical progressor groups, and because we assumed that TPT has no effect on resistant strains, all who did not receive TPT and all who started with resistance to the TPT drug were modeled as eventually progressing to active TB disease.

- 1. Efficacy of preventive therapy against progressive subclinical TB

The estimated effects of TPT on subclinical progressors are a critical set of model inputs. We assumed that some subclinical TB progresses to clinical disease despite TPT (either during the TPT or after its completion), but other cases subclinical TB which would have progressed to clinical disease in absence of TPT is cured by TPT. This is analogous to the effects of TPT on latent TB infections, with are usually but not always cured (i.e. prevented from ever progressing to clinical disease) by TPT. (Meanwhile, a third fraction of subclinical TB, which is not included among the “subclinical progressors”, will resolve before developing symptoms regardless of whether it receives any therapy; this is analogous to latent infection that will never progress to TB disease.)

We assumed that TPT was less effective against subclinical than against latent TB. Specifically, the reduction in efficacy was modeled as a reduction, by a factor σ, in the proportion of progressions to active TB that would be prevented if the TB was subclinical rather than latent at the time of TPT. Thus, we define a subclinical efficacy parameter for 4R (ρ­^s^_4R_ , the relative risk of subclinical progression, comparing 4R to placebo) as

ρ­^s^_4R_ = ρ’_4R +_ σ_4R_(1- ρ’_4R_) .

Direct data to inform the estimate of TPT’s effect on subclinical TB are limited, so we extrapolated from historical data on monotherapy for clinical TB (and apply wide uncertainty bounds):

Reference [24] includes a review of patients treated with 12-month-or-longer regimens in which, due to isoniazid and/or ethambutol resistance, rifampicin (intermittently dosed in some studies) was the only active drug. Of a total of 461 such patients at risk for failure, 67 (14.5%) experienced failure while on therapy; of those who successfully completed treatment and were at risk for relapse, 3.5% (8 of 230) experienced relapse. We take the combined proportion (0.145 + 0.035*(1-0.145) = 0.175) as an approximate upper bound on σ_4R_, noting these symptomatic patients likely had higher bacillary loads and more advanced disease, on average, than is typical of subclinical TB, although the most successful trials used higher rifampicin doses than are prescribed as TPT. The modeled uncertainty includes a wide range centered at 50% of this upper bound value, reflecting a weighted average over the spectrum of bacillary burden in subclinical TB (from smear-positive in roughly one third of patients [51] to paucibacillary disease), and also reflecting uncertainty about the effects of HIV, age, shorter treatment duration, and relative incidence of latent TB progression.

For subclinical progressors treated with 6H, similar data from a 1960 trial of INH vs INH+PAS for active TB [29] shows that approximately 50% of patients treated with INH alone achieved culture conversion and maintained it through 12 months of therapy, while 50% failed. Because of uncertainty in this estimate from a single study, we assume the same reduction in probability of cure, relative to 4R, that was modeled for latent TB: ρ^s^_6H_ = ρ^s^_4R_ + α (1- ρ^s^_4R_).

Parameter estimates are shown in Table S2.

Table S2: Parameter estimates, preventive therapy efficacy and related parameters

| **Parameter** | **Mean estimate** | **Sd** | **Sampled distribution** | **References** |
| --- | --- | --- | --- | --- |
| Odds ratio for incident TB, 4R versus placebo (δ _4R_) | 0.41 | 0.1 | beta | [44] |
| Proportion who progress after 6H, of the future progressors cured by 4R (α); applies to both latent progression and (separately) to subclinical progression | 0.14 | 0.05 | beta | [8, 44] |
| Proportion of incident TB in clinical trial cohorts that results from reinfection after TPT (π) | 0.1 | 0.04 | beta | See above [8, 21] |
| Proportion of TPT recipients who complete enough to experience efficacy effects or resistance risk (τ) | 0.1 | 0.03 | beta | Assumes half of those lost to follow [7, 47, 48] may be cured by a partial course of TPT [49] |
| Loss in 4R TPT efficacy when TB is subclinical rather than latent (σ_4R_) | 0.09 | 0.04 | beta | See above |

- 1. Risk of acquiring resistance when latent, regimen-susceptible TB progresses despite preventive therapy

We estimate the probability of acquiring new resistance if TPT is unsuccessful and an initially susceptible infection progresses to clinical disease. We estimate this first for use of TPT among latent progressors, then among subclinical progressors.

In estimating resistance acquired during preventive therapy of latent TB, we note that essentially all available studies excluded subclinical TB before administering preventive therapy; this is true of recent clinical trials of TPT (which sometimes used microbiological or radiographic screening) and also of historical preventive therapy studies (which used radiographic screening) [8, 52–62].

For 6H and other isoniazid preventive therapy, a meta-analysis [13] estimated an incidence rate ratio of 1.25 for the occurrence of INH-resistant TB after isoniazid preventive therapy versus placebo. (As described above, most of the studies in that review had excluded active TB radiographically prior to TPT.) To convert this incidence rate ratio to a risk of resistance acquisition among those who did not have preexisting resistance, we estimate that 7.4% of TB infections were isoniazid -resistant (estimated global prevalence of isoniazid resistance [63]), and thus that in absence of TPT, 7.4% of incident TB would be isoniazid-resistant. To generate the observed incidence rate ratio of 1.25, and assuming that isoniazid had no effect on preexisting isoniazid -resistant cases, then this corresponds to an incidence of acquired isoniazid resistance equal to 0.25 * 7.4% = 1.85% of the total TB incidence expected after placebo. If we additionally estimate that 6H prevents 50% of all incident TB [44], then the incidence of acquired isoniazid resistance would be equal to 1.85%/(1-50%) = 3.7% of the incident TB that develops despite isoniazid. We use this as the basis for our estimate of acquired isoniazid resistance after 6H.

We initially estimated uncertainty in this estimate based on uncertainty in the observed incidence rate ratio of isoniazid resistant TB (0.75 to 2.1), in the efficacy of 6H (40 to 70% of TB prevented), and in the prevalence of preexisting isoniazid resistance (upper bound 10% at the time of the above trials); this results in a lower bound of zero and an upper bound of 18% of those who progress to TB despite preventive therapy. But a direct examination of the trials included in meta-analysis [13] provides a tighter upper bound: Among placebo recipients, 5.7% developed TB, 30% were testing for resistance, and 8% of these were isoniazid resistant if tested. Applying a binomial distribution with this combined probability and examining the 2.5% quantile as a lower uncertainty bound, we would expect to detect at least qbinom(p = 0.025, size = 18000, prob = 0.057*0.08*0.3, lower.tail = T) = 15 isoniazid-resistant incidence cases among the 18,000 isoniazid recipients even in absence of acquired resistance. Thus, of the 30 resistant cases (out of 158 tested), at least 15 are attributable to preexisting resistance, and an upper bound for the probability of acquired resistance among those without pre-existing resistance is (30-15)/(158-15) = 10%.

For 4R, fewer studies are available for estimating the risk that latent progressors acquire rifampicin resistance, but we used data from clinical trials of rifamycin-based preventive therapy:

- 1. Hong Kong/MRC trial of 3R versus placebo [60]: Incident TB cases included 0 rifampicin resistant cases in the 3R arm (and 2 rifampicin resistant cases in the placebo arm, presumably reflecting preexisting rifampicin-resistant infections), out of 15 and 28 total incident cases in in the respective arms whose susceptibility was determined.
  2. 2018 trial of 4R versus 9H [8]: Incident TB cases included 1 with genotypic rifampicin resistance after 4R, out of 4 bacteriologically-confirmed cases total. If we assume 0.41 odds ratio for incident TB with 4R relative to placebo [44], this corresponds to one rifampicin-resistant case out of ~13 incident bacteriologically-confirmed cases that would have been expected without TPT had there been a placebo arm.
  3. We also considered a 2011 trial of 3HP [64] (the short half-life of isoniazid relative to the weekly dosing interval may make 3HP approach the rifampicin resistance acquisition of rifapentine monotherapy). In this this study, there was 1 rifamycin resistant incident case out of 7 incident cases in the 3HP arm, with ~24 expected cases in absence of TPT if we apply the same estimate of efficacy vs placebo as above.

Combining these trials, there were 2 incident rifampicin resistant TB cases identified in rifampicin-treated arms, out of approximately 65 incident TB cases expected in absence of TPT – rifampicin resistance in 3.1% of the total expected incident cases. The binomial confidence interval on this quantity is wide (approx. 0.5% to >10%), and the estimate requires downward adjustment for expected incidence of rifampicin-resistant TB arising from pre-existing rifampicin resistance, as follows:

The global prevalence of rifampicin resistance is now 3.4% (2.5-4.4%) of all new TB cases [21], but had a weighted average estimate of approximately 2.5% at the times that these trials were performed. Subtracting this background prevalence from the proportion of expected incidence that was rifampicin resistant, we estimate (with wide uncertainty) an acquired rifampicin-resistance incidence equal to 0.6% of the TB incidence that would have been expected in absence of TPT. After adjusting for the estimated 0.29 reduction in TB incidence rate that results from rifampicin TPT, this corresponds to a point estimate of 0.6%/0.29 = 2% for the probability of rifampicin resistance acquisition among all who progress despite 4R. The variance of beta uncertainty distribution was chosen such that the upper 97.5% quantile would be approximately equal to the point estimate of 3.7% for isoniazid, because spontaneous rifampicin resistance arises less frequently than isoniazid resistance and the duration of exposure to 4R is shorter than to 6H.

The acquisition of isoniazid resistance when failing 6H was parametrized as an odds ratio, comparing to the corresponding odds of rifampicin resistance with 6R. The shifted gamma probability distribution for this odds ratio was chosen to have a mean of 2 (based on the point estimates above for the resistance risks associated with 6H and 4R respectively), a lower bound of 1, and a 97.5% quantile of 4.

Parameter estimates are shown in Table S3.

- 1. Risk of acquiring resistance when subclinical, initially-regimen-susceptible TB progresses despite preventive therapy

Although no direct measurements of this quantity exist, potentially relevant data were reviewed, including:

1. In a 1960 INH vs INH+PAS trial for treatment of active TB [29], among the 50% of INH monotherapy patients who failed, 100% developed INH resistance by 6 months. The risk of resistance may be lower, however, for rifampicin therapy (due to rarer spontaneous resistance) and for subclinical TB (due to potentially lower bacillary burden for selective pressure to act upon).
2. In clinical trials of isoniazid- and ethambutol-resistant active TB treated with rifampicin + ethambutol (i.e. effective monotherapy) summarized in [24] and in section 2c above, there were 31 patients who failed and had acquired resistance assessed, of whom 25 (77%) were found to have acquired resistance.
3. Subclinical disease is sometimes paucibacillary. TB cavities contain between 10^7^ and 10^9^ *M tuberculosis* CFU, and a single caseous focus contains ~10^4^ CFU [65]. In comparison, rifampicin resistance occurs spontaneously in 1 per 10^7^ to 10^9^ CFU [66] (i.e., 1 expected resistant organisms per cavity); this is an order of magnitude less common than for INH [67], and suggests that only a small fraction of patients with noncavitary and paucibacillary disease are likely to have spontaneously occurring rifampicin resistance. If pre-existing resistance is present, however, then projections of two-week treatment response data suggest that it is likely to predominate within 2 months of rifampicin monotherapy [66].

Thus, for a subclinical progressor patient population, the risk of acquired resistance with single-drug TPT is likely to depend on the distribution of bacillary burdens among the patients with progressive subclinical disease. For smear-positive patients, the risk is near 100% for INH and >50% for rifampicin. The risk, however, is likely to fall to near zero at intermediate bacillary burdens for rifampicin and at low bacillary burdens for isoniazid.

Considering that 1/3 of subclinical TB in prevalence surveys is smear positive [51, 86] and weighting the probabilities of TPT success accordingly, we estimate an acquired rifampicin resistance risk between 10% (based on the risk of rifampicin resistance acquisition when isoniazid monoresistant active TB fails combination therapy, estimated below), to an upper bound of 50%.

For the risk of INH resistant acquisition when susceptible subclinical TB is inadvertently treated with 6H and progresses, we apply the same odds ratio as for latent TB.

Parameter estimates are shown in Table S3.

Table S3: Parameter estimates, preventive therapy resistance acquisition

| **Parameter** | **Mean estimate** | **SD** | **Sampled distribution** | **References** |
| --- | --- | --- | --- | --- |
| Probability of acquiring rifampicin resistance, if latent rif-S infection progresses despite 4R | 0.02 | 0.008 | beta | [8, 13, 60, 64]; Detailed in section 2d above |
| Probability of acquiring rifampicin resistance, if subclinical rif-S TB progresses despite 4R | 0.28 | 0.1 | beta | [24, 29, 65–67]; Detailed in section 2e above |
| Odd ratio of acquiring new resistance to TPT drug if progresses despite TPT, 6H versus 4R | 2 | 0.5 | Shifted gamma (lower bound 1) | [13]; Detailed in section 2d above |

- 1. Risks of failure or relapse after TB treatment, with or without acquired drug resistance, by initial resistance phenotype

Outcomes of TB disease treatment are modeled as a risk of treatment failure or relapse (both of which count toward the outcome of incident active TB), followed by risks of acquired resistance to isoniazid, rifampicin, or both among those who are not cured by treatment. Parameter estimates are based on previous reviews of clinical trial and research cohort data.

When a standard first-line regimen is used for drug-susceptible TB or for isoniazid mono-resistant TB, the probabilities of cure versus failure/relapse are based on a systematic review [30]. We inflate the probabilities of failure/relapse 1.5x to account for differences between programmatic versus research conditions (including loss to follow up); losses to follow up are categorized based on their expected actual TB outcome (e.g. cure by partial treatment, or relapse or failure after the loss to follow up) rather than their programmatic outcome. For isoniazid mono-resistant TB, we further assume that 20% of patients will have their isoniazid resistance detected and receive a modified regimen [68], improving their probability of treatment success to that modeled for drug-susceptible TB. Among patients with multidrug-resistant TB, we assume that half are bacteriologically positive and receive rifampicin susceptibility testing [21] and an appropriate drug-resistant TB treatment regimen with up to 80% success, while half are treated ineffectively (with 20% success) for presumed drug-susceptible TB. Rifampicin monoresistance (i.e., isoniazid susceptibility, with expected pyrazinamide and ethambutol susceptibility as well) increased the probability of success with first-line therapy when rifampicin resistance was not detected [31].

In estimating the probabilities of acquired resistance among those who are unsuccessfully treated, we use estimates from previous literature reviews [25] to estimate probabilities among those who are initially pan susceptible. The probabilities of acquiring additional (i.e., multidrug) resistance among those with initial drug mono-resistance take into account the different regimens that such patients may receive (with 50% of rifampicin-resistant TB and 80% of isoniazid-mono-resistant TB assumed to receive a standard first-line regimen), the relative risks of failure after each regimen (as above), and the estimated risk of acquiring additional resistance for each possible regimen (estimating that rifampicin-mono-resistant TB has an INH resistance acquisition probability of 85% when failing standard first-line therapy [29] and 0 when receiving or failing drug-resistant TB treatment, and that isoniazid-mono-resistant TB has a rifampicin resistance acquisition probability of 38% (20-60%) when failing a standard first-line regimen [24, 26] and of 20% when failing a modified regimen for INH-resistant TB.

Table S4: Parameter estimates, TB treatment outcomes

| **Parameter** | **Mean estimate** | **SD** | **Sampled distribution** | **References** |
| --- | --- | --- | --- | --- |
| Probability of TB treatment failure/relapse, if initially pan-susceptible | 0.09 | 0.03 | Beta | [30], adjusted as described above |
| Probability of TB treatment failure/relapse, if initially isoniazid mono-resistant | 0.2 | 0.05 | Beta | [30], adjusted as described above |
| Probability of TB treatment failure/relapse, if initially rifampicin mono-resistant | 0.35 | 0.05 | Beta | [31] |
| Probability of TB treatment failure/relapse, if initially multidrug-resistant | 0.5 | 0.35 | Beta | [32] and as described above |
| Probability of rifampicin monoresistance acquisition if treated unsuccessfully and initially pan-susceptible | 0.04 | 0.02 | Beta | [24–28] |
| Probability of isoniazid monoresistance acquisition if treated unsuccessfully and initially pan-susceptible | 0.1 | 0.03 | Beta | [25–28] |
| Probability of multidrug resistance acquisition if treated unsuccessfully and initially pan-susceptible | 0.04 | 0.02 | Beta | [25–28] |
| Probability of multidrug resistance acquisition if treated unsuccessfully and initially rifampicin monoresistant | 0.72 | 0.15 | Beta | [29] and regimen weightings as above |
| Probability of multidrug resistance acquisition if treated unsuccessfully and initially isoniazid monoresistant | 0.31 | 0.1 | Beta | [24, 26] and regimen weightings as above |

- 1. Prevalence of drug resistance:

We use local and national prevalence data to estimate the prevalence of isoniazid and rifampicin resistance among new TB cases. We assume this same prevalence applies to the subclinical progressors and latent progressors within the modeled cohorts. Estimates are shown in table S5.

- 1. Relative sizes of cohort strata

The sizes of each age or CD4 stratum are based on primary data, normalized to a total cohort size of 1000, with resulting numbers in each stratum shown in Table S5.

Table S5: Composition of cohorts with respect to initial drug resistance and age or CD4 count

| **Parameter** | **Setting** | **Subpopulation** | **Mean estimate** | **SD** | **Sampled distribution** | **References** |
| --- | --- | --- | --- | --- | --- | --- |
| Subpopulation size | KZN | CD4<100 | 126 |  | NA | Primary data |
| Subpopulation size | KZN | CD4 100-200 | 147 |  | NA | **“** |
| Subpopulation size | KZN | CD4 200-350 | 262 |  | NA | **“** |
| Subpopulation size | KZN | CD4 >350 | 464 |  | NA | **“** |
| Subpopulation size | Pakistan | Age <5yo | 167 |  | NA | **“** |
| Subpopulation size | Pakistan | Age 5-15yo | 325 |  | NA | **“** |
| Subpopulation size | Pakistan | Age >15yo | 507 |  | NA | **“** |
| Prevalence of isoniazid monoresistance among infected TPT recipients (i.e. proportion of future TB progressions that will be monoresistant, if no TPT) | KZN |  | 0.05 | 0.015 | Beta | [69] |
| Prevalence of rifampicin monoresistance among infected TPT recipients | KZN |  | 0.007 | .015 | Beta | [70] |
| Prevalence of multidrug resistance among infected TPT recipients | KZN |  | 0.027 | .004 | Beta | [21] |
| Prevalence of isoniazid monoresistance among infected TPT recipients | Pakistan |  | 0.079 | .015 | Beta | [63] (≤ global average because excluding known INH-R TB contacts) |
| Prevalence of rifampicin monoresistance among infected TPT recipients | Pakistan |  | 0.001 | .0005 | Beta | [71, 72] |
| Prevalence of multidrug resistance among infected TPT recipients | Pakistan |  | 0.019 | .005 | Beta | [63] (reduced 50% because excluding DR-TB contacts) |

- 1. Sensitivity, access, and requirements of subclinical TB screening

We compare scenarios with screening for subclinical TB (e.g. by chest x-ray or sputum Xpert) prior to TPT, with scenarios that screen only by symptoms.

Primary analyses assume that screening, if performed, has 90% sensitivity for detecting subclinical progressors. This is consistent with the overall sensitivity of chest X-ray or Xpert Ultra to detect culture-positive TB [73, 74]; the proportion of subclinical progressors detected by these modalities may be lower to the extent that it is less advanced or more paucibacillary on average, but use of this estimate reflects the fact that the cases most likely to be missed by these screening modalities are more likely than average to be curable with preventive therapy.

We also consider scenarios in which a requirement for subclinical TB screening reduces access to TPT. We assume that all who undergo symptom screening and are eligible for TPT will be prescribed TPT (100% access), and that a requirement for additional testing may reduce access to TPT. Specifically, a proportion between 20 to 100% of the patient population does not complete the required subclinical TB screening and as a result is neither diagnosed with subclinical TB (if they have it) nor offered TPT.

1. State transition steps:

At the start of the model, the cohort is stratified by age (HHCs) or CD4 count (PWH); by TB state (latent progressor, subclinical progressor, or other, in proportions that vary by stratum); and by drug susceptibilities (pan-susceptible, isoniazid-monoresistant, rifampicin-monoresistant, or multidrug-resistant, in the same proportions for all latent and subclinical progressor strata in a given setting).

The cohort then transitions to initial outcomes after the TPT step, as follows:

- In the no-TPT scenario, all latent and subclinical progressors are designated as eventually developing active TB (modeled outcomes do not depend on the timing of that progression), with drug susceptibilities as initially assigned.
- In the TPT (4R or 6H) scenarios with no subclinical TB screening, all undergo TPT, with outcomes of cure or eventual progression depending on the initial TB state, TPT regimen, and initial drug susceptibilities. For those who eventually progress but not have resistance to the TPT drug at baseline, there is a risk of acquiring drug resistance during TPT that depends on the TPT regimen and initial TB state.
- In the TPT (4R or 6H) scenarios with screening for subclinical TB, a proportion of the cohort lose access to the intervention due to the screening requirement, and they experience the same outcomes as in the no-TPT scenario. Of the remainder, all latent progressors and those subclinical progressor whose disease is not detected (due to imperfectly sensitive tests) experience the same outcomes as in the corresponding TPT scenario without screening. Finally, the subclinical progressors whose disease is diagnosed receive treatment; this treatment results in outcomes of either cure or non-cure (i.e., failure or future relapse), and those not cured may acquire new or additional drug resistance (depending on the regimen and the initial drug susceptibilities).

Finally, the cohort transitions to outcomes after a possible future round of TB treatment

- For those who were cured by TPT or by initial treatment for subclinical disease, no further TB events are modeled.
- For those who did not receive TPT or treatment, or whose TPT or treatment was not successful at preventing eventual progression to active disease, a round of treatment is modeled. The outcome of treatment is either cure or failure to cure, and for those who are not cured, there is a possibility of acquiring resistance; the probabilities of non-cure and of acquired resistance both depend on the drug susceptibilities at the intiation of this treatment course.

1. Probabilistic parameter sampling:

Parameter estimates were represented as a distribution – beta distributions when bounded by 0 and 1, gamma when bounded below by zero only, and a shifted gamma when bounded below by a value other than 1. Mean values were estimated as described above for each parameter, with standard deviations chosen based either on the desired variance or a particular desired quantile. A latin hypercube was used to sample from all parameters’ estimated distributions simultaneously for each run of the model.

SUPPLEMENTAL RESULTS

Table S6. Correspondence between baseline culture-positive TB prevalence and 3-month clinical TB incidence in cohort of people with newly diagnosed HIV and a negative TB symptom screen in Kwa-Zulu Natal.

|  | **CD4 <100** | **CD4 100-200** | **CD4 200-350** | **CD4 >350** |
| --- | --- | --- | --- | --- |
| Included in culture sub-study, N | 52 | 81 | 100 | 192 |
| Culture positive at enrollment, N (%) | 18 (35%  [23-48%]) | 9 (11%  [6-20%]) | 7 (7%  [3-14%]) | 9 (5%  [2-9%]) |
| Not included in culture sub-study, N | 327 | 361 | 685 | 1200 |
| Diagnosed with TB within 3 months, not in sub-study, N (%) | 106 (32%  [28-38%]) | 68 (19%  [15-23%]) | 70 (10%  [8-13%]) | 83 (7%  [6-9%]) |

Figure S1: Projected outcomes of 4R or 6H, each compared to no TPT, among a cohort of 1000 all-age Household contacts.

Panel A shows results with no screening for subclinical TB prior to TPT, and panel B shows results with screening for subclinical TPT under the assumption that it reduces access to TPT by 20%.

**
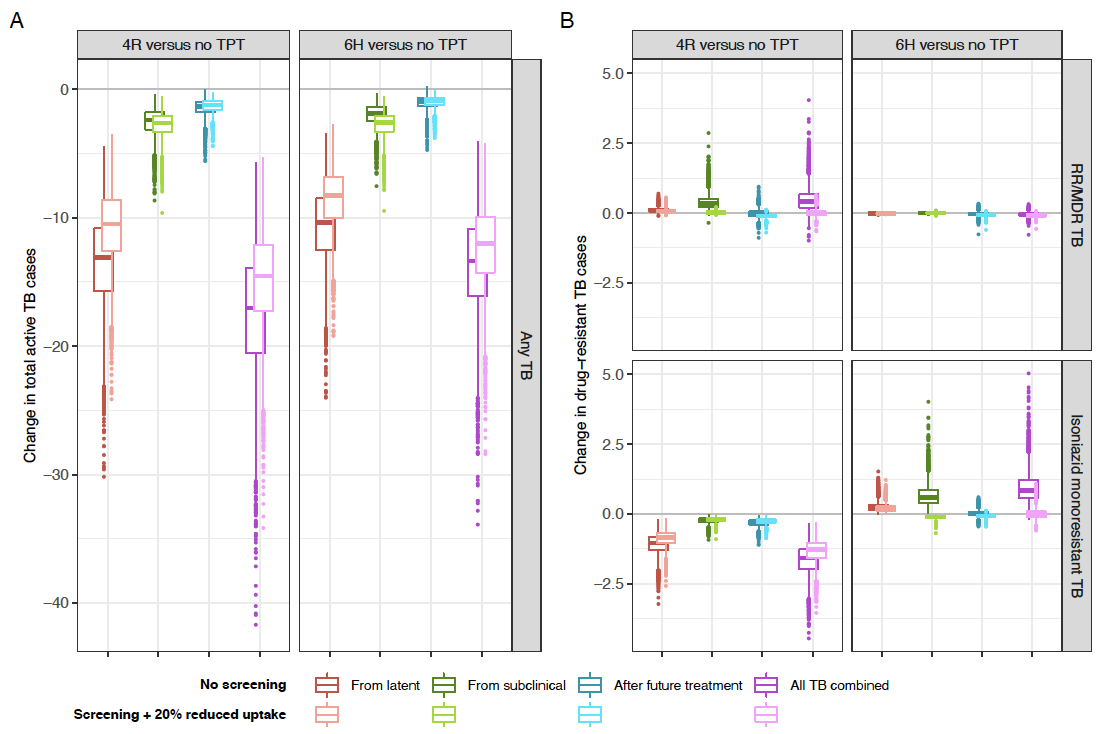
**

Figure S2: Head-to-head comparison of TB outcomes after TPT, comparing 4R regimen to 6H

Negative values indicate fewer cases with 4R than with 6H.


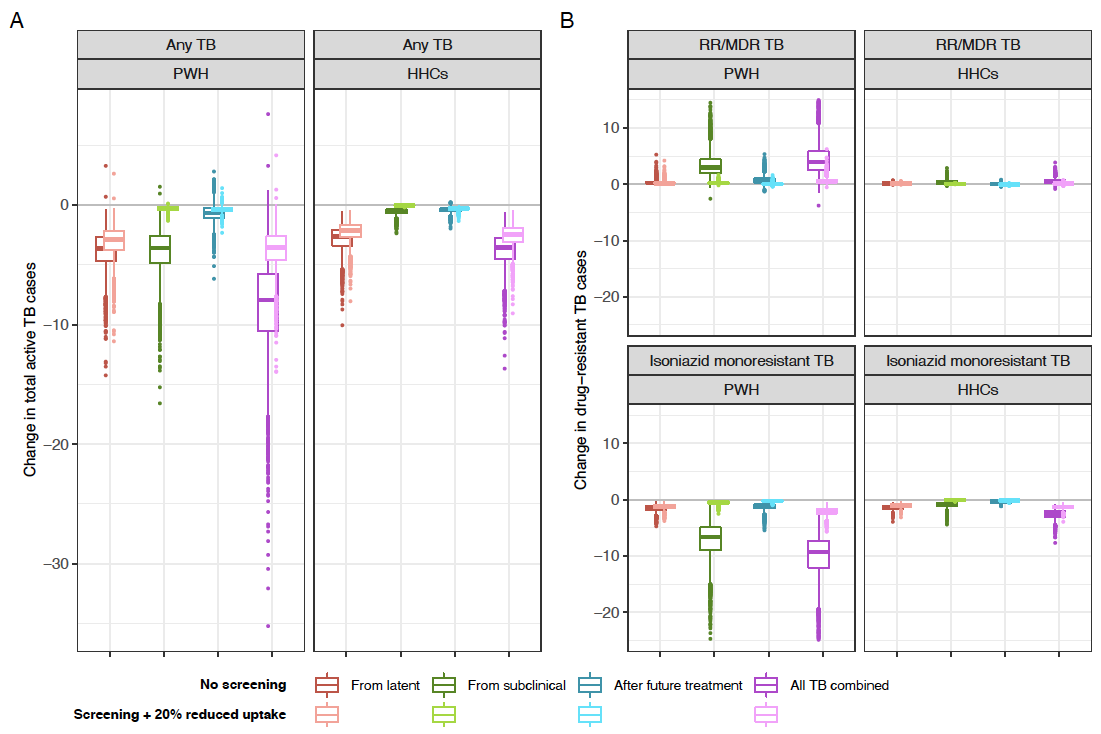


Figure S3: Effect of reduced access on the impact of subclinical TB screening prior to TPT, among HHC cohort

**
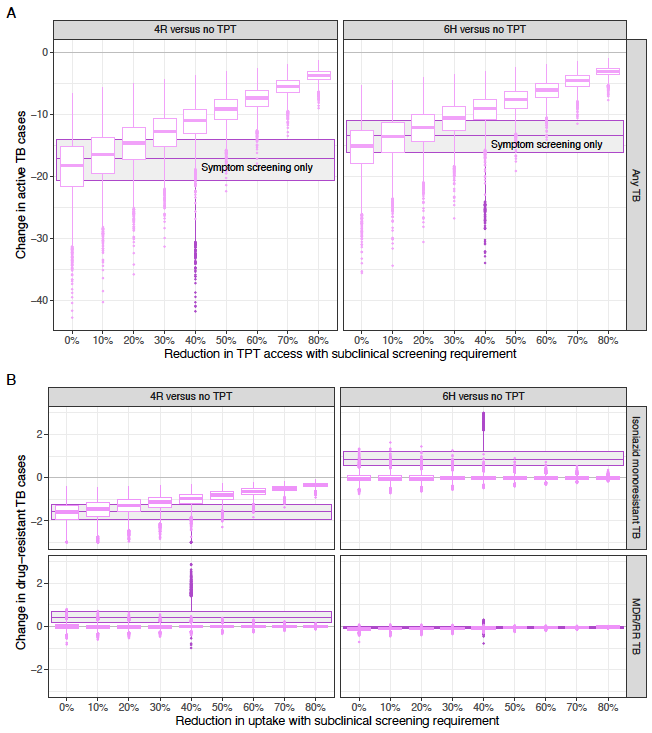
**

Figure S4: Sensitivity of key results to individual parameters. For each parameter, the 10% of simulations that used the highest values of that parameter are compared to the 10% that used the lowest values. Gray bars show the difference in median values of each outcome between these two subsets of simulations, and the error bars show the interquartile range of the outcome within each subset (blue for the highest decile of values for that parameter, red for the lowest decile).


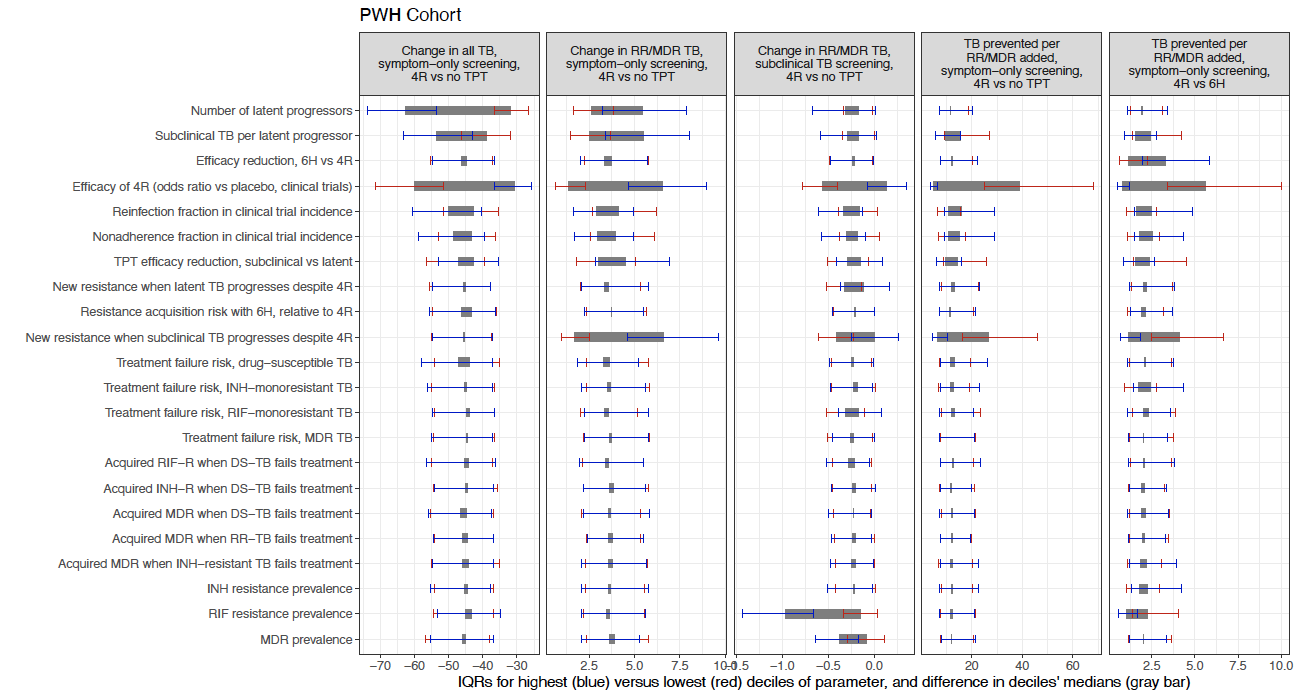

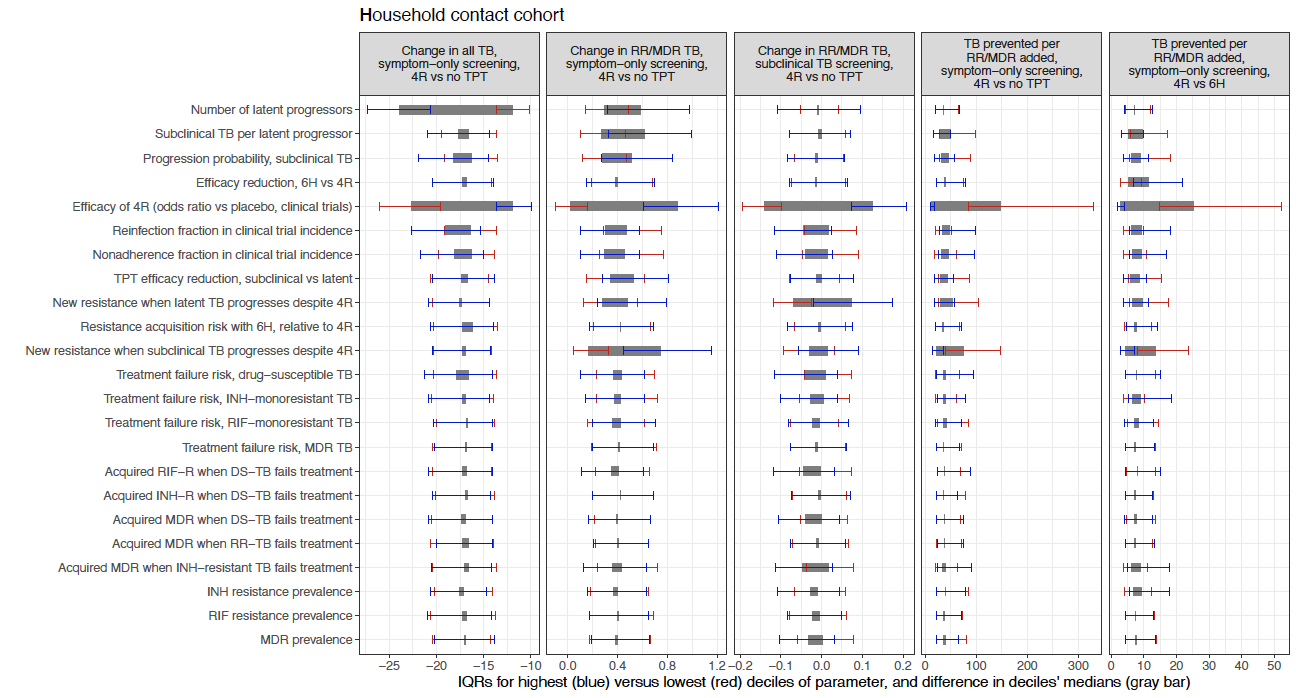


Figure S5: Projected outcomes of 4R or 6H, each compared to no TPT, among 1000 household contacts, when TPT is only considered for the 17% of contacts who are under age 5


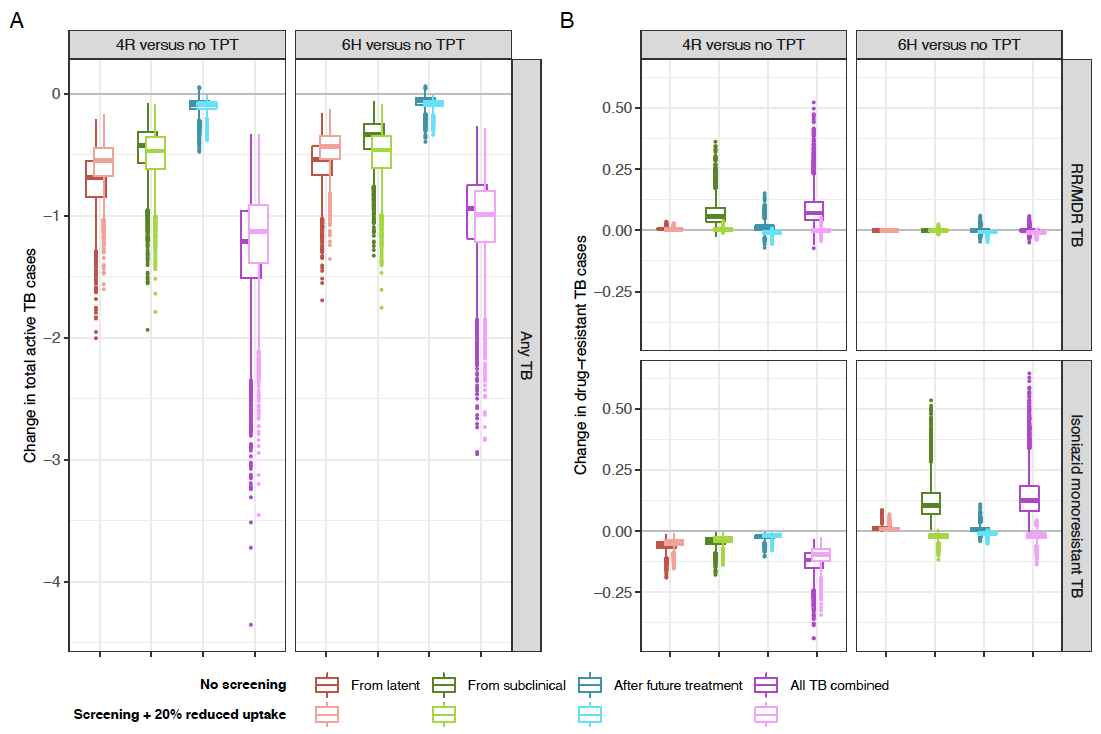

Supplement: Supplementary file 1 — Additional file 1: Supplemental methods, Tables S1-S6, and Figures S1-S5. Supplemental methods – Additional details of primary human subjects data collection, estimation of model parameters, state-transition model, and approach to probabilistic parameter sampling. Table S1. Parameter estimates, sizes of latent and subclinical progressor populations. Table S2. Parameter estimates, preventive therapy efficacy and related parameters. Table S3. Parameter estimates, preventive therapy resistance acquisition. Table S4. Parameter estimates, TB treatment outcomes. Table S5. Composition of cohorts with respect to initial drug resistance and age or CD4 count. Table S6. Correspondence between baseline culture-positive TB prevalence and 3-month clinical TB incidence in cohort of people with newly diagnosed HIV and a negative TB symptom screen in Kwa-Zulu Natal. Fig S1. – Projected outcomes of 4R or 6H, each compared to no TPT, among a cohort of 1000 all-age Household contacts. Fig S2. Head-to-head comparison of TB outcomes after TPT, comparing 4R regimen to 6H. Fig S3. Effect of reduced access on the impact of subclinical TB screening prior to TPT, among HHC cohort. Fig S4. Sensitivity of key results to individual parameters. Fig S5 – Projected outcomes of 4R or 6H, each compared to no TPT, among 1000 household contacts, when TPT is only considered for the 17% of contacts who are under age 5 [file 12916_2021_2189_MOESM1_ESM.docx]
